# Molecular detection of *Cercopithifilaria*, *Cruorifilaria* and *Dipetalonema*-like filarial nematodes in ticks of French Guiana[Fn FN1]

**DOI:** 10.1051/parasite/2023027

**Published:** 2023-07-04

**Authors:** Florian Binetruy, Olivier Duron

**Affiliations:** MIVEGEC, University of Montpellier (UM), Centre National de la Recherche Scientifique (CNRS), Institut pour la Recherche de la Développement (IRD) 34394 Montpellier France

**Keywords:** Cercopithifilaria, Cruorifilaria, Dipetalonema, Amblyomma, Ixodes, Rhipicephalus

## Abstract

Filarial nematodes of the *Dipetalonema* lineage are widespread parasites and include some species that are transmitted by ticks. In this study, we conducted a large molecular survey of ticks in French Guiana, South America, to understand the overall diversity of tick-borne filarioids in this remote region largely covered by dense tropical forests. Out of 682 ticks belonging to 22 species and 6 genera, 21 ticks (3.1%) of the species *Amblyomma cajennense*, *A. oblongoguttatum*, *A. romitii*, *Ixodes luciae* and *Rhipicephalus sanguineus sensu lato* were positive for infection by filarioids. Molecular typing and phylogenetic analysis identified all these filarioids as members of the *Dipetalonema* lineage. While the filarioid of *R. sanguineus sensu lato* is a previously described species, the canine worm *Cercopithifilaria bainae* Almeida & Vicente, 1984, all other filarioids detected in this study are related but distinct to already known species in the genera *Cercopithifilaria*, *Cruorifilaria* and *Dipetalonema*. Their vertebrate host range may include a wide variety of mammals present in French Guiana, but dogs, capybaras, and opossums are the best candidate hosts for some of these filarioids. Although the detection of members of the *Dipetalonema* lineage in ticks of significant medical or veterinary interest is of concern, the risk of contracting a tick-borne filarial infection is still largely unknown. The pathogenicity of these filarioids, their epidemiology, developmental cycles, and mechanisms of transmission by South American tick species now require further study.

## Abstract (fr)

Les nématodes filaires de la lignée *Dipetalonema* sont des parasites répandus dont plusieurs espèces sont transmises par les tiques. Dans cette étude, nous avons mené une vaste surveillance moléculaire des tiques en Guyane française, en Amérique du Sud, afin de caractériser la diversité des filaires transmis par les tiques dans cette région largement couverte de forêts tropicales denses. Sur 682 tiques appartenant à 22 espèces et 6 genres, 21 tiques (3.1 %) des espèces *Amblyomma cajennense*, *A. oblongoguttatum*, *A. romitii*, *Ixodes luciae* et *Rhipicephalus sanguineus sensu lato* étaient positives pour la détection des filaires. Le typage moléculaire et l’analyse phylogénétique ont permis d’identifier toutes ces filaires comme des membres de la lignée *Dipetalonema*. Alors que la filaire de *R. sanguineus sensu lato* est une espèce décrite, la filaire canine *Cercopithifilaria bainae* Almeida & Vicente, 1984, toutes les autres filaires détectées ici sont apparentées mais distinctes des espèces déjà connues au sein des genres *Cercopithifilaria*, *Cruorifilaria* et *Dipetalonema*. Leur spectre d’hôtes vertébrés pourrait inclure une grande variété de mammifères présents en Guyane française, mais les chiens, les capibaras et les opossums sont les hôtes candidats probables pour certaines de ces filaires. Bien que la détection de membres de la lignée *Dipetalonema* chez des tiques d’intérêt médical ou vétérinaire soit préoccupante, le risque de contracter une filariose à tiques est encore largement inconnu. La pathogénicité de ces filaires à tiques, leur épidémiologie, leurs cycles de développement et les mécanismes de transmission par les espèces de tiques sud-américaines doivent maintenant être étudiés plus en détail.

## Introduction

Ticks are important vectors of viruses, bacteria, and protozoan parasites of medical and veterinary importance [31, 44]. Ticks can also harbor filarial nematodes of the family Onchocercidae Leiper, 1911, commonly known as filariae or filarioids, but their presence is not often the focus of research on tick-borne pathogens [10, 13, 45, 46, 56]. Members of the Onchocercidae family have evolved specialized parasitic life cycles in terrestrial vertebrates and in a variety of blood feeding dipterans (mosquitoes, biting midges, sand flies, horse flies, black flies, etc.) which are the main vectors for most species [29, 46, 64, 67]. In vertebrate hosts, mature filarioids live in the circulatory system or in particular tissues and release microfilariae into the bloodstream. The microfilariae are next taken up by blood-feeding arthropod vectors in which they develop into infective larvae that can be transmitted to a new vertebrate host through further biting. Some filarioids are causative agents of major human (lymphatic filariasis, onchocerciasis, loiasis) and animal diseases (canine heartworm disease) [29, 64, 67].

Early microscopic morphological observations provided evidence for the presence of filarioids in field populations of ticks: Filarioids have been morphologically identified in North America in *Ixodes cookei* [4] and *I. dammini* [12], in South America in *Amblyomma cajennense* [74] and *Ornithodoros talaje* [37], in Europe in *I. ricinus* [1, 60, 73] and *Rhipicephalus sanguineus* sensu lato (*s.l.*) [7], in North Africa in *R. sanguineus s.l.* [5], in Australia in *I. trichosuri* [66] and in Western Asia in *O. tartakowskyi* [11]. More recently, filarioids were also morphologically identified in Japan in *Haemaphysalis flava* and *H. japonica* [71]. Molecular investigations confirmed the presence of filarioids in *I. ricinus* [60] and *R. sanguineus s.l.* [13, 45, 56], and further led to their detection in other tick species in North America, *I. scapularis* [30, 51, 69, 70] and *Amblyomma americanum* [40, 76]. Field surveys demonstrated that infection rates of filarioids can reach 5% in some populations of *I. ricinus* [1, 60], and may be as high as 25% in *I. scapularis* [51] and 50% in some populations of *R. sanguineus s.l.* [13, 45].

Microscopic observations and molecular typing converge to identify most filarioids of ticks as members of the genera *Acanthocheilonema* Cobbold, 1870, *Monanema* Anteson, 1968, *Yatesia* Bain, Baker & Chabaud, 1982, and *Cercopithifilaria* Eberhard, 1980 [1, 5, 7, 11, 12, 40, 46, 51, 56, 59, 60, 69, 71]. While early microscopic morphological observations classified some filarioids of ticks as members of the genus *Dipetalonema* Diesing, 1861, numerous subgenera or species formerly included in this genus were further renamed and elevated to generic rank as *Cercopithifilaria* [8, 10, 28]. Phylogenetic analyses based on molecular and morphological data further showed that the genera *Acanthocheilonema*, *Monanema*, *Yatesia* and *Cercopithifilaria* (all associated with ticks), as well as the genera *Dipetalonema* (associated with biting midges), *Litomosoides* Chandler, 1931 (associated with parasitic mites), and *Cruorifilaria* Eberhard, Morales & Orihel, 1976 (not yet associated with a vector), cluster in a monophyletic clade of filarioids, termed as the *Dipetalonema* lineage or the ONC4 clade, within the family Onchocercidae [8, 10, 28, 46]. Some members of the *Dipetalonema* lineage could provide immunologically relevant and experimentally tractable laboratory models of human onchocerciasis since they cause skin and ocular immunopathologies in vertebrates analogous to those observed in humans infected with *Onchocerca volvulus* Leuckart, 1893 [66, 72].

Experimental infection assays have confirmed the vector competence of ticks for filarioids of the genera *Acanthocheilonema*, *Monanema*, *Yatesia* and *Cercopithifilaria*. Baltazard *et al.* [11] first experimentally showed that the soft tick *O. tartakovskyi* can be infected by *Acanthocheilonema viteae* (Krepkogorskaya, 1933; formerly *Dipetalonema viteae*) after feeding on infected field vertebrates and that this tick species can further transmitted viable infective larvae to domestic rodents through biting. This tick-borne infection cycle has been conducted for several decades in the laboratory using *O. tartakovskyi* as an exclusive vector [5, 6, 48], or *O. moubata* as an alternative vector [63]. Further experimental infection assays showed similar vector competence in pairing of hard ticks (belonging to the genera *Ixodes*, *Rhipicephalus*, *Amblyomma*, *Haemaphysalis* and *Hyalomma*) and some filarioids of the *Dipetalonema* lineage: ticks feeding on infected vertebrates ingest microfilariae, which can develop up to the viable infective third stage in a few weeks and are further excreted with saliva during biting [4, 6, 9, 14–16, 24, 42, 52, 53, 58, 66, 73, 74]. Filarioids of the *Dipetalonema* lineage also survive transstadially in such ticks since the development from microfilariae to infective third-stage larvae occurs only while the tick is off-host, that is, during ecdysis from tick larva to nymph or from nymph to adult [53, 66]. While all these observations converge to confirm that ticks are effective vectors for these filarioids, members of the *Dipetalonema* lineage are currently neglected tick-borne parasites [10, 46, 56].

In this study, we conducted a molecular survey of filarioids in ticks in French Guiana. This remote territory is a vast equatorial land located on the north–east coast of South America, mostly covered by dense rainforests and with a low density of human population [68]. We know little on the presence and diversity of tick-borne diseases in French Guiana, but recent surveys led to the description of novel tick-borne pathogens [17, 21, 32, 43]. Here, we surveyed 682 field ticks belonging to 22 tick species of the 33 known from French Guiana [19]. We further used cytochrome c oxidase subunit I (*cox1*) mitochondrial gene sequences, and phylogenetics for the description of filarioid infections in these ticks. Lastly, we examined and discussed their genetic proximity with already known species of filarioids associated with ticks.

## Materials and methods

### Tick collection

Field ticks (*n* = 682; belonging to 22 tick species) were collected in French Guiana either on vegetation by the drag-flag method (for questing ticks) or directly from their vertebrate hosts or seabird nests (for engorged or questing ticks) (Tables 1 and S1). Samples included five major tick genera belonging to the family Ixodidae (hard ticks): *Amblyomma* (16 species), *Rhipicephalus* (2 species), *Ixodes* (1 species), *Dermacentor* (1 species) and *Haemaphysalis* (1 species). Samples include one species of the *Ornithodoros* genus, belonging to the Argasidae family (soft ticks). All ticks were stored in 75% ethanol until examination. Ticks were further examined under a Leica Z16 APO A macroscope and categorized by species, stage and sex in previous studies [18–20]. Use of genetic resources was approved by the French Ministry of the Environment under reference #TREL19028117S/156, in compliance with the Access and Benefit Sharing procedure implemented by the *Loi pour la Reconquête de la Biodiversité*.

**Table 1:** List of tick species examined for infection by filarioids in French Guiana (cf. Table S1 for more details).

Host species	Number of examined specimens	Number of infected specimens (%)
Argasidae (soft ticks)		
*Ornithodoros capensis* Neumann, 1901	6	0 (0)
Ixodidae (hard ticks)		
*Amblyomma cajennense* (Fabricius, 1787)	229	15 (6.6%)
*Amblyomma calcaratum* Neumann, 1899	1	0 (0)
*Amblyomma coelebs* Neumann, 1899	31	0 (0)
*Amblyomma dissimile* Koch, 1884	21	0 (0)
*Amblyomma geayi* Neumann, 1899	10	0 (0)
*Amblyomma goeldii* Neumann, 1899	5	0 (0)
*Amblyomma humerale* Koch, 1844	10	0 (0)
*Amblyomma latepunctatum* Tonelli-Rondelli, 1939	4	0 (0)
*Amblyomma longirostre* (Koch, 1844)	137	0 (0)
*Amblyomma naponense* (Packard, 1869)	5	0 (0)
*Amblyomma oblongoguttatum* Koch, 1844	66	2 (3%)
*Amblyomma pacae* Aragão, 1911	6	0 (0)
*Amblyomma romitii* Tonelli-Rondelli, 1939	2	1 (50%)
*Amblyomma rotundatum* Koch, 1844	6	0 (0)
*Amblyomma scalpturatum* Neumann, 1906	9	0 (0)
*Amblyomma varium* Koch, 1844	7	0 (0)
*Rhipicephalus microplus* (Canestrini, 1888)	10	0 (0)
*Rhipicephalus sanguineus s.l.* (Latreille, 1806)	6	1 (16.7%)
*Dermacentor nitens* Neumann, 1897	97	0 (0)
*Haemaphysalis juxtakochi* Cooley, 1946	8	0 (0)
*Ixodes luciae* Senevet, 1940	6	2 (33.3%)
All tick species	682	21 (3.1%)

### Molecular detection and typing of filarioids in ticks

DNA was extracted from individual ticks using a DNeasy Blood & Tissue Kit (QIAGEN, Hilden, Germany), following the manufacturer’s instructions. Each individual extract was then tested by a polymerase chain reaction (PCR) assay for filarioid infection by amplifying a fragment of the *cox1* mitochondrial gene (689 bp) using the CO1intF (5′–TGA TTG GTG GTT TTG GTA A–3′) and CO1intR (5′–ATA AGT ACG AGT ATC AAT ATC–3′) primers [26]. PCRs were performed in a total volume of 25 μL containing 10–50 ng of genomic DNA, 8 mM of each dNTP (Thermo Fisher Scientific, Waltham, MA, USA), 10 mM of MgCl_2_ (Thermo Fisher Scientific), 7.5 μM of each of the internal primers, 2.5 μL of 10× PCR buffer (Thermo Fisher Scientific), and 1.25 U of Taq DNA polymerase (Thermo Fisher Scientific). All PCR amplifications were performed as follows: initial denaturation at 93 °C for 3 min, 35 cycles of denaturation (93 °C, 30 s), annealing (50 °C, 30 s), extension (72 °C, 1 min), and a final extension (72 °C, 5 min). Positive (DNA of the filarioid *Loa loa*) and negative (water) controls were included in each PCR assay. Following visualization via electrophoresis in 1.5% agarose gel, positive PCR products were sequenced by Eurofins. Sequence chromatograms were cleaned with Chromas Lite (http://www.technelysium.com.au/chromas_lite.html), and alignments were performed using ClustalW, implemented in the MEGA software package (https://www.megasoftware.net/). New sequences obtained in this study were deposited in GenBank under accession numbers OR030075–OR030095.

### Molecular phylogenetic analyses

Phylogenetic analyses were based on sequence alignments of the filarioid *cox1* gene sequences obtained in this study. Sequences of other filarioids obtained from GenBank, including representative members of the *Dipetalonema* lineage (*Acanthocheilonema*, *Yatesia*, *Cercopithifilaria*, *Cruorifilaria*, *Litomosoides* and *Dipetalonema*) and of other filarial nematodes were also included in the phylogenetic analyses. The Basic Local Alignment Search Tool (BLAST; https://blast.ncbi.nlm.nih.gov/blast/Blast.cgi) was used to find additional sequences available on GenBank and showing the highest nucleotide similarities with the filarioid gene sequences we characterized in this study. The Gblocks program with default parameters was used to obtain non-ambiguous sequence alignments [27]. Tree-based phylogenetic analyses were performed using maximum-likelihood (ML) analyses using the MEGA software package (https://www.megasoftware.net/). The evolutionary models that best fit the sequence data were determined using the Akaike information criterion. Clade robustness was assessed by bootstrap analysis using 1,000 replicates.

### Statistical analyses

All statistical analyses were carried out using the R statistical package (https://www.r-project.org/).

## Results

### Detection of filarioids in ticks

Among the 682 tick specimens from French Guiana, *cox1* PCR assay indicated the presence of filarioids in 21 ticks (3.1%) belonging to five species: *A. cajennense* (*n* = 15 infected specimens), *A. oblongoguttatum* (*n* = 2), *A. romitii* (*n* = 1), *I. luciae* (*n* = 2) and *R. sanguineus s.l.* (*n* = 1) (Tables 1 and S1). No filarioid was detected in the 17 other tick species. The detection rate of filarioids did not co-vary with the screening effort, i.e., the number of examined specimens per tick species (Spearman’s rank correlation, *r*_*s*_ = 0.24, *p* = 0.28): The tick species observed with filarioids were not always among those for which we examined more specimens. This was shown by *A. romitii* (*n* = 2 examined specimens, 1 filarioid-positive specimen: 50%), *I. luciae* (*n* = 6 examined specimens, 2 filarioid-positive specimens: 33.3%) and *R. sanguineus s.l.* (*n* = 6 examined specimens, 1 filarioid-positive specimens: 16.7%), although we detected filarioid-positive specimens in tick species for which we examined a large number of specimens: *A. cajennense* (*n* = 229 examined specimens, 15 filarioid-positive specimens: 6.6%) and *A. oblongoguttatum* (*n* = 66 examined specimens, two filarioid-positive specimens: 3%). Filarioids were not detected in some tick species for which we examined a large number of specimens, such as *A. longirostre* (*n* = 137 examined specimens but none positive) and *Dermacentor nitens* (*n* = 97 examined specimens but none positive) (Tables 1 and S1). Of the 21 filarioid-infected tick specimens, 18 were unfed *A. cajennense* (*n* = 15), *A. oblongoguttatum* (*n* = 2) and *R. sanguineus s.l.* (*n* = 1) ticks while three were engorged *A. romitii* (*n* = 1) and *I. luciae* (*n* = 2) ticks collected on capybaras and opossums, respectively (Table S1). Of the 21 filarioid-infected tick specimens, 12 were *A. cajennense* nymphs and nine were adults (*A. cajennense*, *n* = 3 including one male and two females; *A. oblongoguttatum*, *n* = 2 females; *A. romitii*, *n* = 1 female; *I. luciae*, *n* = 2 including one male and one female; *R. sanguineus s.l.*, *n* = 1 male) (Table S1).

### Molecular typing of filarioids in ticks

On the basis of *cox1* gene sequences, we characterized six genetically distinct filarioids (pairwise nucleotide identity: 84.1-99.8%) in *A. cajennense*, *A. oblongoguttatum*, *A. romitii*, *I. luciae* and *R. sanguineus s.l*. Two genetically distinct filarioids were detected in *A. cajennense* (pairwise nucleotide identity: 87.1%), two in *I. luciae* (pairwise nucleotide identity: 99.5%), and one in each of the other infected tick species (*A. oblongoguttatum*, *A. romitii* and *R. sanguineus s.l.*). Of the six genetically distinct filarioids, each was specific to its respective tick species, expect one which was shared by *A. cajennense* and *I. luciae*.

The filarioid *cox1* gene sequence we detected in *R. sanguineus s.l.* was identical to those of *Cercopithifilaria bainae* Almeida & Vicente, 1984, previously characterized from dogs in Italy and the USA (GenBank accession numbers: KF270686, KP760175, MH390716; 100% pairwise nucleotide identities) [23, 46, 65]. However, none of the filarioid *cox1* gene sequences we identified in *A. cajennense*, *A. oblongoguttatum*, *A. romitii* and *I. luciae* was 100% identical to other filarioid sequences available on GenBank, although showing high levels of nucleotide identity with some members of the *Dipetalonema* lineage. The filarioid *cox1* gene sequence of *A. oblongoguttatum* showed highest pairwise nucleotide identities with members of the genus *Cercopithifilaria*, including *Cercopithifilaria bainae* infecting dogs (GenBank accession numbers: KF270686, KP760175, MH390716; 87.1% pairwise nucleotide identities) [23, 46, 65], *Cercopithifilaria grassii* (Noe, 1907) infecting dogs (JQ837810; 88.7%) [57], *Cercopithifilaria* sp. II sensu Otranto et al., 2013 [55] infecting dogs (KX898457; 87.9%) [49] and *Cercopithifilaria multicauda* Uni & Bain, 2001, infecting Japanese serow (AB178848; 87.6%) [2]. The filarioid *cox1* gene sequence of *A. romitii* and one of *A. cajennense* showed highest pairwise nucleotide identities with *Cruorifilaria tuberocauda* Eberhard, Morales & Orihel,1976, infecting capybara (KP760176; 98.7-90.1%) [46]. The two filarial *cox1* gene sequences of *I. luciae* and one of *A. cajennense* showed highest pairwise nucleotide identities with members of the genus *Dipetalonema* infecting monkeys, including *Dipetalonema yatesi* Notarnicola, Jiménez & Gardner, 2007 (MW199183; 88%) [75], *Dipetalonema gracile* (Rudolphi, 1809) (KP760181; 88.9%) [46] and *Dipetalonema caudispina* (Molin, 1858) (MT502200; 88.9%) [62].

### Phylogeny of filarioids in ticks

**Figure 1:**
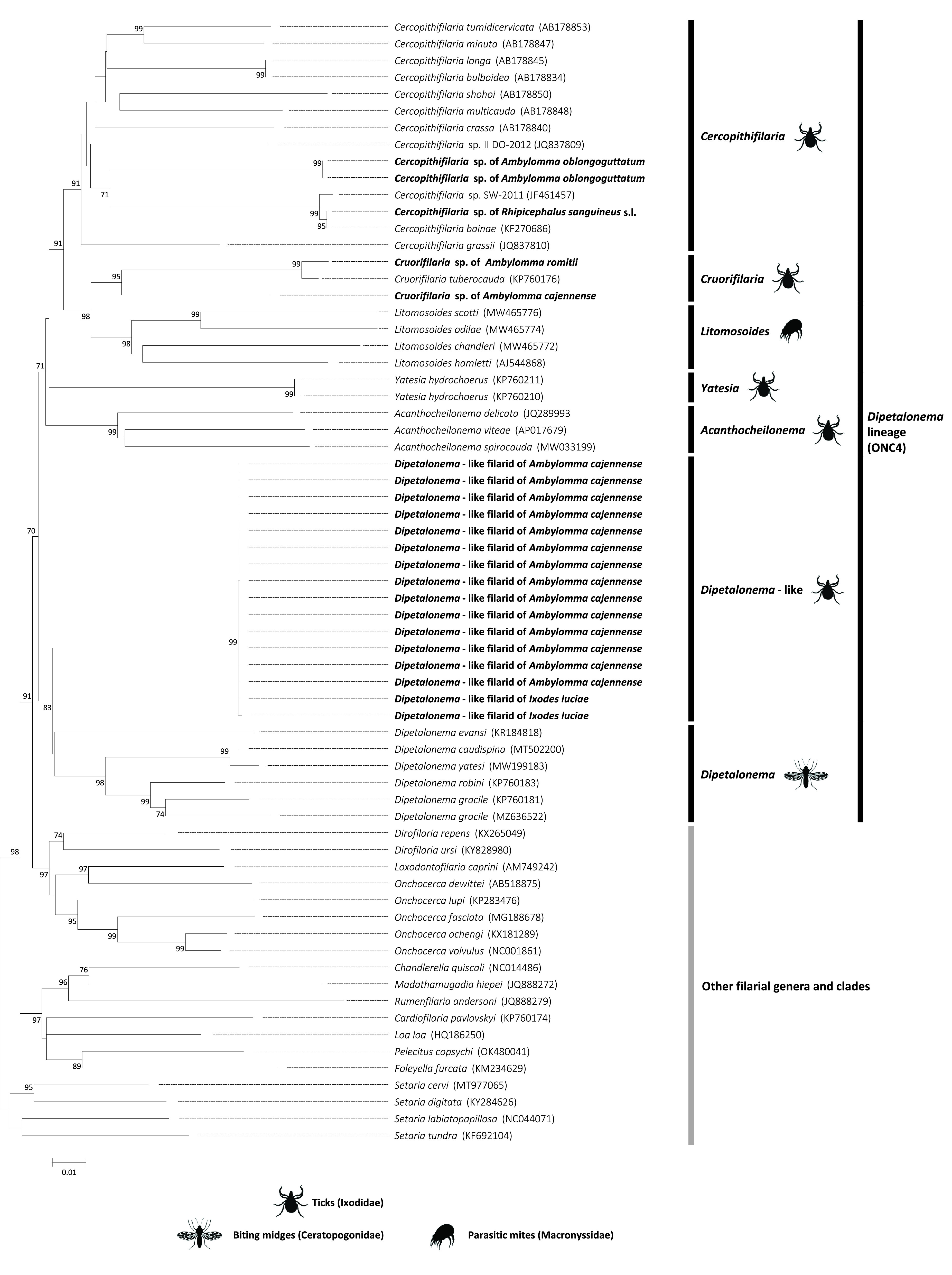
Phylogeny of Onchocercid filarioids constructed using maximum-likelihood (ML) estimations based on *cox1* nucleotide sequences with a total of 649 unambiguously aligned bp (best-fit approximation for the evolutionary model: GTR+G+I). Major genera of the *Dipetalonema* lineage (*Acanthocheilonema*, *Yatesia*, *Cercopithifilaria*, *Cruorifilaria*, *Litomosoides* and *Dipetalonema*), including representative species with indication of vector range (ticks, biting midges, parasitic mites), are indicated. Filarioid sequences obtained in this study from the ticks *A. cajennense* (*n* = 15), *A. oblongoguttatum* (*n* = 2), *A. romitii* (*n* = 2), *I. luciae* (*n* = 2) and *R. sanguineus s.l.* (*n* = 1) are in bold. GenBank accession numbers of sequences used in analyses are shown on the phylogenetic trees. Numbers at nodes indicate percentage support of 1,000 bootstrap replicates. Only bootstrap values >70% are shown. The scale bar is in units of substitution/site.

A ML analysis based on *cox1* nucleotide sequences was further conducted to examine the phylogenetic proximity between the filarioids of *A. cajennense*, *A. oblongoguttatum*, *A. romitii*, *I. luciae* and *R. sanguineus s.l.* and with other filarioids. Phylogenetic reconstruction revealed a clustering of the genera *Cercopithifilaria*, *Cruorifilaria*, *Litomosoides*, *Yatesia*, *Acanthocheilonema* and *Dipetalonema* in a unique monophyletic clade, the *Dipetalonema* lineage, distinct to other members of the family Onchocercidae (Fig. 1). Phylogenetic reconstruction further showed within the *Dipetalonema* lineage that:

1. The filarioids of *A. oblongoguttatum* and *R. sanguineus s.l.* formed a robust subclade, supported by a 91% bootstrap value, together with all species of the genus *Cercopithifilaria* (Fig. 1). This suggested that the filarioids of *A. oblongoguttatum* and *R. sanguineus s.l.* belong to the genus *Cercopithifilaria*. While the filarioid of *R. sanguineus s.l.* was probably *Cercopithifilaria bainae* (as suggested by their 100% pairwise nucleotide identity), the filarioid of *A. oblongoguttatum* was distinct from already known *Cercopithifilaria* species.
2. The filarioid of *A. romitii* and one of *A. cajennense* delineated a robust subclade (95% bootstrap value) together with *Cruorifilaria tuberocauda* (Fig. 1), suggesting that all belong to the genus *Cruorifilaria*. The filarioid of *A. romitii* and one of *A. cajennense* were distinct to already known *Cruorifilaria* species.
3. The other filarioid of *A. cajennense* and those of *I. luciae* clustered in a unique robust subclade (provisionally named *Dipetalonema*-like hereafter; 99% bootstrap value) substantially divergent from all other genera of the *Dipetalonema* lineage (Fig. 1). The phylogenetic proximity of this filarioid of *A. cajennense* to those of *I. luciae*, and their 99.8–100% pairwise nucleotide identities, suggested that they probably belong to the same species. The closest relative of *Dipetalonema*-like is the genus *Dipetalonema*, while other genera are more distantly related. This suggests that these *Dipetalonema*-like filarioids may belong to a potential novel genus which could be a sister genus of *Dipetalonema*.

## Discussion

We show here that substantial diversity of filarioids of the *Dipetalonema* lineage is present in ticks of French Guiana. We conducted extensive molecular screening of 22 tick species, and identified filarioids in five tick species, *A. cajennense*, *A. oblongoguttatum*, *A. romitii*, *I. luciae* and *R. sanguineus s.l.* No filarioid was detected in the 17 other tick species. While pairwise nucleotide identities and phylogenetics assigned the filarioid of *R. sanguineus s.l.* to an already described species, *Cercopithifilaria bainae*, all other filarioids detected in this study are distinct to already known species of *Cercopithifilaria*, *Cruorifilaria* and *Dipetalonema* on the basis of their *cox1* gene sequences. To our knowledge, this is also the first observation of *Cruorifilaria* in ticks (and, more broadly, in blood-feeding arthropods), suggesting that *A. cajennense* and *A. romitii* are vectors of this filarioid genus.

The presence of filarioid DNA in *A. cajennense*, *A. oblongoguttatum*, *A. romitii*, *I. luciae* and *R. sanguineus s.l.* suggests that these tick species may serve as vectors for filarial nematodes. Obviously, these observations are not conclusive evidence of vector competence: ticks feeding on parasitemic vertebrates can be positive for filarioid DNA due to the presence of infected blood in the tick digestive tract, but may not be competent to transmit infectious filarioids during their next blood meal. However, experimental infection assays have directly confirmed the vector competence of *Ixodes*, *Amblyomma* and *Rhipicephalus* ticks for filarioids of the *Dipetalonema* lineage, including the brown dog tick *R. sanguineus s.l.* for *Cercopithifilaria bainae* [4, 6, 24, 42, 52, 53, 58, 66, 73, 74]. In the field, the detection of viable microfilariae and infective filarial larvae in the hindgut, haemocoele, fat cells or ducts of salivary glands of ticks also support these observations [5, 7, 12]. In addition, the accumulation of microfilariae in the dermis of infected vertebrates at the sites of attachment of ticks suggest a specific chemotactic response of filarioids to tick saliva [42, 50]. Furthermore, our detection of filarioids from questing (unfed) *A. cajennense*, *A. oblongoguttatum*, and *R. sanguineus s.l.* ticks that have already digested their previous blood meals, have further moulted, and are seeking vertebrates for their next blood meal suggests that these tick species can acquire and maintain filarioids of the *Dipetalonema* lineage, a pattern of transstadial transmission also observed for other tick species [53, 66]. Finally, the observation that all tick-associated filarioids cluster in a monophyletic clade, the *Dipetalonema* lineage, distinct from clades of filarioids specifically associated with transmission by blood-feeding dipterans, supports the conclusion that ticks are vectors of these filarioids.

Tick-borne filarioids have been detected from most continents, suggesting worldwide distribution of the *Dipetalonema* lineage [10, 46]. Some species may have global distribution such as *Cercopithifilaria bainae*, specifically associated with dogs and *R. sanguineus s.l.*, which was first formally identified in Brazil [3], and further in Europe [41, 56, 65], Asia [13, 45], Iran [61], North America [23, 47], and now in French Guiana (this study). The other filarioids we characterized in French Guiana have not been detected anywhere and their transmission cycles remain unknown. In fact, the vertebrate hosts of the *Cercopithifilaria* sp. of *A. oblongoguttatum* remain unidentified since this tick is a generalist species feeding on a wide range of mammals [19].

For the *Cruorifilaria* spp. of *A. romitii* and *A. cajennense*, two biological features suggest that capybara could be their vertebrate host. First, the single described *Cruorifilaria* species, *Cruorifilaria tuberocauda*, has been reported as a parasite of capybara in other South American regions [25, 33], and, second, *A. romitii* is a tick species highly specific to capybara, while *A. cajennense* is a generalist tick species often found on these animals [19]. For the *Dipetalonema*-like filarioids, opossums could be vertebrate hosts since one of the infected tick species, *I. luciae*, is a specialized tick species that feeds primarily on opossums [19]. Past studies have morphologically identified four filarial species, all of the *Dipetalonema* lineage, in South American opossums: *Acanthocheilonema pricei* (Vaz et Pereira, 1934), *Cercopithifilaria didelphis* (Esslinger et Smith, 1979), *Skrjabinofilaria skrjabini* Travassos, 1925, and *Cherylia guyanensis* Bain et al., 1985 [9, 35]. No molecular data are currently available for these four species, which prevents any definitive conclusions.

The detection of members of the *Dipetalonema* lineage in ticks of significant medical or veterinary interest is of concern, but the risk of acquiring a tick-borne filarial infection is unknown. While *A. cajennense* and *A. oblongoguttatum* are the tick species most commonly biting humans in South America [36, 39], in the present study, we found that 3–6.6% of field specimens were infected with filarioids of the *Dipetalonema* lineage. Humans are therefore exposed to bites of filarioid-infected ticks without the risk of infection being documented to date. In North America, a survey of humans with a tick-bite history for the presence of antibodies against the *I. scapularis*-associated filarioid found, however, no evidence of infection [69]. In animals, filarioids of the *Dipetalonema* lineage are often considered innocuous, but some studies and evidence suggest that they are not. In dogs, recent surveys showed that *Cercopithifilaria bainae* can induce erythematous and pruritic dermatitis, subcutaneous nodules, ulcerative skin lesions and chronic polyarthritis [23, 38, 54]. Infections of dogs by other members of the *Dipetalonema* lineage, *Acanthocheilonema* spp., were also associated with clinical symptoms, such as anemia and high levels of plasma proteins [22, 34]. In capybaras, adults of *Cruorifilaria tuberocauda* form tangled masses in the kidneys, and less frequently in the pulmonary arteries, where they cause extensive tissue damage with severe endarteritis, leading to obstruction of vessels and infarction of adjacent tissues [33].

In conclusion, we have identified a wide range of *Cercopithifilaria*, *Cruorifilaria* and *Dipetalonema*-like filarial nematodes in French Guiana. The repeated detection of the *Dipetalonema* lineage in major tick genera on most continents confirms that these are widespread but neglected tick-borne filarioids of largely undocumented veterinary (and perhaps medical) concern. Their pathogenicity, epidemiology, developmental cycles and transmission mechanisms by ticks must be further investigated.

## Supplementary Materials

The supplementary material of this article is available at https://www.parasite-journal.org/10.1051/parasite/2023027/olm. *Table S1*: Details of tick species and specimens examined for infection by filarioids in French Guiana.
