# Supplementary material for: Molecular detection of Cercopithifilaria, Cruorifilaria and Dipetalonema-like filarial nematodes in ticks of French Guiana
Source: Parasite. 2023 Jul 4;30:24. doi: 10.1051/parasite/2023027 (PMC10321233; doi:10.1051/parasite/2023027)
Supplement: Supplementary file 1 — Table S1: Details of tick species and specimens examined for infection by filarioids in French Guiana. [file parasite-30-24-s1.pdf]

SUPPLEMENTARY MATERIAL

Molecular detection of *Cercopithifilaria* , *Cruorifilaria* and *Dipetalonema* -like filarial nematodes in ticks of French Guiana

Florian Binetruy and Olivier Duron

Table S1. Details of tick species and specimens examined for infection by filarioids in French Guiana.

Host species	Species local name	Family	Sampling location	Sampling date	Stage	Sex	Feeding status	Filarioid (0: non-infected; 1: infected)
Ornithodoros capensis	Seabird tick	Argasidae	Grand Connétable Island	2016	Adult	Male	Engorged tick, collected in a laughing gull nest	0
Ornithodoros capensis	Seabird tick	Argasidae	Grand Connétable Island	2016	Adult	Male	Engorged tick, collected in a laughing gull nest	0
Ornithodoros capensis	Seabird tick	Argasidae	Grand Connétable Island	2016	Nymph	_	Engorged tick, collected in a laughing gull nest	0
Ornithodoros capensis	Seabird tick	Argasidae	Grand Connétable Island	2016	Nymph	_	Engorged tick, collected in a laughing gull nest	0
Ornithodoros capensis	Seabird tick	Argasidae	Grand Connétable Island	2016	Adult	Female	Engorged tick, collected in a laughing gull nest	0
Ornithodoros capensis	Seabird tick	Argasidae	Grand Connétable Island	2016	Adult	Female	Engorged tick, collected in a laughing gull nest	0
Amblyomma cajennense	Cayenne tick	Ixodidae	Kourou (CSG)	2016	Adult	Female	Questing tick, collected on human	0
Amblyomma cajennense	Cayenne tick	Ixodidae	Kourou (CSG)	2016	Adult	Female	Questing tick, collected on human	0
Amblyomma cajennense	Cayenne tick	Ixodidae	Kourou (CSG)	2016	Adult	Female	Questing tick, collected on human	0
Amblyomma cajennense	Cayenne tick	Ixodidae	Kourou (CSG)	2016	Adult	Female	Questing tick, collected on human	0
Amblyomma cajennense	Cayenne tick	Ixodidae	Kourou (CSG)	2016	Adult	Female	Questing tick, collected on human	1
Amblyomma cajennense	Cayenne tick	Ixodidae	Kourou (CSG)	2016	Adult	Female	Questing tick, collected on human	0
Amblyomma cajennense	Cayenne tick	Ixodidae	Kourou (CSG)	2016	Adult	Female	Questing tick, collected on human	0
Amblyomma cajennense	Cayenne tick	Ixodidae	Kourou (CSG)	2016	Adult	Female	Questing tick, collected on human	0
Amblyomma cajennense	Cayenne tick	Ixodidae	Kourou (CSG)	2016	Adult	Female	Questing tick, collected on human	0
Amblyomma cajennense	Cayenne tick	Ixodidae	Kourou (CSG)	2016	Adult	Female	Questing tick, collected on human	0
Amblyomma cajennense	Cayenne tick	Ixodidae	Kourou (CSG)	2016	Adult	Female	Questing tick, collected on human	0
Amblyomma cajennense	Cayenne tick	Ixodidae	Kourou (CSG)	2016	Adult	Female	Questing tick, collected on human	0
Amblyomma cajennense	Cayenne tick	Ixodidae	Kourou (CSG)	2016	Adult	Female	Questing tick, collected on human	0
Amblyomma cajennense	Cayenne tick	Ixodidae	Kourou (CSG)	2016	Adult	Female	Questing tick, collected on human	0
Amblyomma cajennense	Cayenne tick	Ixodidae	Kourou (CSG)	2016	Adult	Female	Questing tick, collected on human	0
Amblyomma cajennense	Cayenne tick	Ixodidae	Kourou (CSG)	2016	Adult	Female	Questing tick, collected on human	0
Amblyomma cajennense	Cayenne tick	Ixodidae	Kourou (CSG)	2016	Adult	Female	Questing tick, collected on human	0
Amblyomma cajennense	Cayenne tick	Ixodidae	Kourou (CSG)	2016	Adult	Female	Questing tick, collected on human	0
Amblyomma cajennense	Cayenne tick	Ixodidae	Kourou (CSG)	2016	Adult	Female	Questing tick, collected on human	0
Amblyomma cajennense	Cayenne tick	Ixodidae	Kourou (CSG)	2016	Adult	Female	Questing tick, collected on human	0
Amblyomma cajennense	Cayenne tick	Ixodidae	Kourou (CSG)	2016	Adult	Female	Questing tick, collected on human	0
Amblyomma cajennense	Cayenne tick	Ixodidae	Kourou (CSG)	2016	Adult	Female	Questing tick, collected on human	0
Amblyomma cajennense	Cayenne tick	Ixodidae	Kourou (CSG)	2016	Adult	Female	Questing tick, collected on human	0
Amblyomma cajennense	Cayenne tick	Ixodidae	Kourou (CSG)	2016	Adult	Female	Questing tick, collected on human	0
Amblyomma cajennense	Cayenne tick	Ixodidae	Kourou (CSG)	2016	Adult	Female	Questing tick, collected on human	0
Amblyomma cajennense	Cayenne tick	Ixodidae	Kourou (CSG)	2016	Adult	Female	Questing tick, collected on human	0
Amblyomma cajennense	Cayenne tick	Ixodidae	Kourou (CSG)	2016	Adult	Female	Questing tick, collected on human	0
Amblyomma cajennense	Cayenne tick	Ixodidae	Kourou (CSG)	2016	Adult	Female	Questing tick, collected on human	0
Amblyomma cajennense	Cayenne tick	Ixodidae	Kourou (CSG)	2016	Adult	Female	Questing tick, collected on human	0
Amblyomma cajennense	Cayenne tick	Ixodidae	Kourou (CSG)	2016	Adult	Female	Questing tick, collected on human	0
Amblyomma cajennense	Cayenne tick	Ixodidae	Kourou (CSG)	2016	Adult	Male	Questing tick, collected on human	0
Amblyomma cajennense	Cayenne tick	Ixodidae	Kourou (CSG)	2016	Adult	Male	Questing tick, collected on human	0
Amblyomma cajennense	Cayenne tick	Ixodidae	Kourou (CSG)	2016	Adult	Male	Questing tick, collected on human	0
Amblyomma cajennense	Cayenne tick	Ixodidae	Kourou (CSG)	2016	Adult	Male	Questing tick, collected on human	0
Amblyomma cajennense	Cayenne tick	Ixodidae	Kourou (CSG)	2016	Adult	Male	Questing tick, collected on human	0
Amblyomma cajennense	Cayenne tick	Ixodidae	Kourou (CSG)	2016	Adult	Male	Questing tick, collected on human	0
Amblyomma cajennense	Cayenne tick	Ixodidae	Kourou (CSG)	2016	Adult	Male	Questing tick, collected on human	0
Amblyomma cajennense	Cayenne tick	Ixodidae	Kourou (CSG)	2016	Adult	Male	Questing tick, collected on human	0
Amblyomma cajennense	Cayenne tick	Ixodidae	Kourou (CSG)	2016	Adult	Male	Questing tick, collected on human	0
Amblyomma cajennense	Cayenne tick	Ixodidae	Kourou (CSG)	2016	Adult	Male	Questing tick, collected on human	0
Amblyomma cajennense	Cayenne tick	Ixodidae	Kourou (CSG)	2016	Adult	Male	Questing tick, collected on human	0
Amblyomma cajennense	Cayenne tick	Ixodidae	Kourou (CSG)	2016	Adult	Male	Questing tick, collected on human	0
Amblyomma cajennense	Cayenne tick	Ixodidae	Kourou (CSG)	2016	Adult	Male	Questing tick, collected on human	0
Amblyomma cajennense	Cayenne tick	Ixodidae	Kourou (CSG)	2016	Adult	Male	Questing tick, collected on human	0
Amblyomma cajennense	Cayenne tick	Ixodidae	Kourou (CSG)	2016	Adult	Male	Questing tick, collected on human	0
Amblyomma cajennense	Cayenne tick	Ixodidae	Kourou (CSG)	2016	Adult	Male	Questing tick, collected on human	0
Amblyomma cajennense	Cayenne tick	Ixodidae	Kourou (CSG)	2016	Adult	Male	Questing tick, collected on human	0
Amblyomma cajennense	Cayenne tick	Ixodidae	Kourou (CSG)	2016	Adult	Male	Questing tick, collected on human	0
Amblyomma cajennense	Cayenne tick	Ixodidae	Kourou (CSG)	2016	Adult	Male	Questing tick, collected on human	0
Amblyomma cajennense	Cayenne tick	Ixodidae	Kourou (CSG)	2016	Adult	Male	Questing tick, collected on human	0
Amblyomma cajennense	Cayenne tick	Ixodidae	Kourou (CSG)	2016	Adult	Male	Questing tick, collected on human	0
Amblyomma cajennense	Cayenne tick	Ixodidae	Kourou (CSG)	2016	Adult	Male	Questing tick, collected on human	0
Amblyomma cajennense	Cayenne tick	Ixodidae	Kourou (CSG)	2016	Adult	Male	Questing tick, collected on human	0
Amblyomma cajennense	Cayenne tick	Ixodidae	Kourou (CSG)	2016	Adult	Male	Questing tick, collected on human	0
Amblyomma cajennense	Cayenne tick	Ixodidae	Kourou (CSG)	2016	Adult	Male	Questing tick, collected on human	0
Amblyomma cajennense	Cayenne tick	Ixodidae	Kourou (CSG)	2016	Adult	Male	Questing tick, collected on human	0
Amblyomma cajennense	Cayenne tick	Ixodidae	Kourou (CSG)	2016	Adult	Male	Questing tick, collected on human	0
Amblyomma cajennense	Cayenne tick	Ixodidae	Kourou (CSG)	2016	Adult	Male	Questing tick, collected on human	0
Amblyomma cajennense	Cayenne tick	Ixodidae	Kourou (CSG)	2016	Adult	Male	Questing tick, collected on human	0
Amblyomma cajennense	Cayenne tick	Ixodidae	Kourou (CSG)	2016	Adult	Male	Questing tick, collected on human	0
Amblyomma cajennense	Cayenne tick	Ixodidae	Kourou (CSG)	2016	Nymph	_	Questing tick, collected on human	0
Amblyomma cajennense	Cayenne tick	Ixodidae	Kourou (CSG)	2016	Nymph	_	Questing tick, collected on human	0
Amblyomma cajennense	Cayenne tick	Ixodidae	Kourou (CSG)	2016	Nymph	_	Questing tick, collected on human	0
Amblyomma cajennense	Cayenne tick	Ixodidae	Kourou (CSG)	2016	Nymph	_	Questing tick, collected on human	0
Amblyomma cajennense	Cayenne tick	Ixodidae	Kourou (CSG)	2016	Nymph	_	Questing tick, collected on human	0
Amblyomma cajennense	Cayenne tick	Ixodidae	Kourou (CSG)	2016	Nymph	_	Questing tick, collected on human	1
Amblyomma cajennense	Cayenne tick	Ixodidae	Kourou (CSG)	2016	Nymph	_	Questing tick, collected on human	0
Amblyomma cajennense	Cayenne tick	Ixodidae	Kourou (CSG)	2016	Nymph	_	Questing tick, collected on human	0
Amblyomma cajennense	Cayenne tick	Ixodidae	Kourou (CSG)	2016	Nymph	_	Questing tick, collected on human	0
Amblyomma cajennense	Cayenne tick	Ixodidae	Kourou (CSG)	2016	Nymph	_	Questing tick, collected on human	0
Amblyomma cajennense	Cayenne tick	Ixodidae	Kourou (CSG)	2016	Nymph	_	Questing tick, collected on human	0
Amblyomma cajennense	Cayenne tick	Ixodidae	Kourou (CSG)	2016	Nymph	_	Questing tick, collected on human	0
Amblyomma cajennense	Cayenne tick	Ixodidae	Petit-Saut (Montagne Plomb)	2016	Nymph	_	Questing tick, collected on vegetation	0
Amblyomma cajennense	Cayenne tick	Ixodidae	Petit-Saut (Montagne Plomb)	2016	Nymph	_	Questing tick, collected on vegetation	0
Amblyomma cajennense	Cayenne tick	Ixodidae	Petit-Saut (Montagne Plomb)	2016	Nymph	_	Questing tick, collected on vegetation	0
Amblyomma cajennense	Cayenne tick	Ixodidae	Petit-Saut (Montagne Plomb)	2016	Nymph	_	Questing tick, collected on vegetation	0
Amblyomma cajennense	Cayenne tick	Ixodidae	Petit-Saut (Montagne Plomb)	2016	Nymph	_	Questing tick, collected on vegetation	0
Amblyomma cajennense	Cayenne tick	Ixodidae	Petit-Saut (Montagne Plomb)	2016	Nymph	_	Questing tick, collected on vegetation	0
Amblyomma cajennense	Cayenne tick	Ixodidae	Petit-Saut (Montagne Plomb)	2016	Nymph	_	Questing tick, collected on vegetation	0
Amblyomma cajennense	Cayenne tick	Ixodidae	Petit-Saut (Montagne Plomb)	2016	Nymph	_	Questing tick, collected on vegetation	0
Amblyomma cajennense	Cayenne tick	Ixodidae	Petit-Saut (Montagne Plomb)	2016	Nymph	_	Questing tick, collected on vegetation	0
Amblyomma cajennense	Cayenne tick	Ixodidae	Petit-Saut (Montagne Plomb)	2016	Nymph	_	Questing tick, collected on vegetation	0
Amblyomma cajennense	Cayenne tick	Ixodidae	Petit-Saut (Montagne Plomb)	2016	Nymph	_	Questing tick, collected on vegetation	0
Amblyomma cajennense	Cayenne tick	Ixodidae	Petit-Saut (Montagne Plomb)	2016	Nymph	_	Questing tick, collected on vegetation	0
Amblyomma cajennense	Cayenne tick	Ixodidae	Petit-Saut (Montagne Plomb)	2016	Nymph	_	Questing tick, collected on vegetation	0
Amblyomma cajennense	Cayenne tick	Ixodidae	Petit-Saut (Montagne Plomb)	2016	Nymph	_	Questing tick, collected on vegetation	0
Amblyomma cajennense	Cayenne tick	Ixodidae	Petit-Saut (Montagne Plomb)	2016	Nymph	_	Questing tick, collected on vegetation	0
Amblyomma cajennense	Cayenne tick	Ixodidae	Petit-Saut (Montagne Plomb)	2016	Nymph	_	Questing tick, collected on vegetation	0
Amblyomma cajennense	Cayenne tick	Ixodidae	Petit-Saut (Montagne Plomb)	2016	Nymph	_	Questing tick, collected on vegetation	0
Amblyomma cajennense	Cayenne tick	Ixodidae	Petit-Saut (Montagne Plomb)	2016	Nymph	_	Questing tick, collected on vegetation	0
Amblyomma cajennense	Cayenne tick	Ixodidae	Petit-Saut (Montagne Plomb)	2016	Nymph	_	Questing tick, collected on vegetation	0
Amblyomma cajennense	Cayenne tick	Ixodidae	Petit-Saut (Montagne Plomb)	2016	Nymph	_	Questing tick, collected on vegetation	0
Amblyomma cajennense	Cayenne tick	Ixodidae	Petit-Saut (Montagne Plomb)	2016	Nymph	_	Questing tick, collected on vegetation	0
Amblyomma cajennense	Cayenne tick	Ixodidae	Petit-Saut (Montagne Plomb)	2016	Larva	_	Questing tick, collected on vegetation	0
Amblyomma cajennense	Cayenne tick	Ixodidae	Matoury (Piste de la Mirande)	2016	Nymph	_	Questing tick, collected on vegetation	0
Amblyomma cajennense	Cayenne tick	Ixodidae	Matoury (Piste de la Mirande)	2016	Nymph	_	Questing tick, collected on vegetation	0
Amblyomma cajennense	Cayenne tick	Ixodidae	Matoury (Piste de la Mirande)	2016	Nymph	_	Questing tick, collected on vegetation	0
Amblyomma cajennense	Cayenne tick	Ixodidae	Matoury (Piste de la Mirande)	2016	Nymph	_	Questing tick, collected on vegetation	0
Amblyomma cajennense	Cayenne tick	Ixodidae	Matoury (Piste de la Mirande)	2016	Nymph	_	Questing tick, collected on vegetation	1
Amblyomma cajennense	Cayenne tick	Ixodidae	Matoury (Piste de la Mirande)	2016	Nymph	_	Questing tick, collected on vegetation	1
Amblyomma cajennense	Cayenne tick	Ixodidae	Matoury (Piste de la Mirande)	2016	Nymph	_	Questing tick, collected on vegetation	0
Amblyomma cajennense	Cayenne tick	Ixodidae	Matoury (Piste de la Mirande)	2016	Nymph	_	Questing tick, collected on vegetation	1
Amblyomma cajennense	Cayenne tick	Ixodidae	Matoury (Piste de la Mirande)	2016	Nymph	_	Questing tick, collected on vegetation	0
Amblyomma cajennense	Cayenne tick	Ixodidae	Matoury (Piste de la Mirande)	2016	Nymph	_	Questing tick, collected on vegetation	0
Amblyomma cajennense	Cayenne tick	Ixodidae	Matoury (Piste de la Mirande)	2016	Nymph	_	Questing tick, collected on vegetation	1
Amblyomma cajennense	Cayenne tick	Ixodidae	Matoury (Piste de la Mirande)	2016	Nymph	_	Questing tick, collected on vegetation	1
Amblyomma cajennense	Cayenne tick	Ixodidae	Matoury (Piste de la Mirande)	2016	Nymph	_	Questing tick, collected on vegetation	1
Amblyomma cajennense	Cayenne tick	Ixodidae	Matoury (Piste de la Mirande)	2016	Nymph	_	Questing tick, collected on vegetation	0
Amblyomma cajennense	Cayenne tick	Ixodidae	Matoury (Piste de la Mirande)	2016	Nymph	_	Questing tick, collected on vegetation	1
Amblyomma cajennense	Cayenne tick	Ixodidae	Matoury (Piste de la Mirande)	2016	Nymph	_	Questing tick, collected on vegetation	1
Amblyomma cajennense	Cayenne tick	Ixodidae	Matoury (Piste de la Mirande)	2016	Nymph	_	Questing tick, collected on vegetation	0
Amblyomma cajennense	Cayenne tick	Ixodidae	Matoury (Piste de la Mirande)	2016	Nymph	_	Questing tick, collected on vegetation	1
Amblyomma cajennense	Cayenne tick	Ixodidae	Matoury (Piste de la Mirande)	2016	Nymph	_	Questing tick, collected on vegetation	0
Amblyomma cajennense	Cayenne tick	Ixodidae	Matoury (Piste de la Mirande)	2016	Nymph	_	Questing tick, collected on vegetation	0
Amblyomma cajennense	Cayenne tick	Ixodidae	Matoury (Piste de la Mirande)	2016	Nymph	_	Questing tick, collected on vegetation	0
Amblyomma cajennense	Cayenne tick	Ixodidae	Matoury (Piste de la Mirande)	2016	Nymph	_	Questing tick, collected on vegetation	0

Amblyomma cajennense	Cayenne tick	Ixodidae	Matoury (Piste de la Mirande)	2016	Nymph	_	Questing tick, collected on vegetation	0
Amblyomma cajennense	Cayenne tick	Ixodidae	Matoury (Piste de la Mirande)	2016	Nymph	_	Questing tick, collected on vegetation	1
Amblyomma cajennense	Cayenne tick	Ixodidae	Matoury (Piste de la Mirande)	2016	Nymph	_	Questing tick, collected on vegetation	0
Amblyomma cajennense	Cayenne tick	Ixodidae	Matoury (Piste de la Mirande)	2016	Nymph	_	Questing tick, collected on vegetation	0
Amblyomma cajennense	Cayenne tick	Ixodidae	Matoury (Piste de la Mirande)	2016	Nymph	_	Questing tick, collected on vegetation	0
Amblyomma cajennense	Cayenne tick	Ixodidae	Matoury (Piste de la Mirande)	2016	Nymph	_	Questing tick, collected on vegetation	1
Amblyomma cajennense	Cayenne tick	Ixodidae	Matoury (Piste de la Mirande)	2016	Nymph	_	Questing tick, collected on vegetation	0
Amblyomma cajennense	Cayenne tick	Ixodidae	Matoury (Piste de la Mirande)	2016	Adult	Female	Questing tick, collected on vegetation	0
Amblyomma cajennense	Cayenne tick	Ixodidae	Matoury (Piste de la Mirande)	2016	Adult	Female	Questing tick, collected on vegetation	0
Amblyomma cajennense	Cayenne tick	Ixodidae	Matoury (Piste de la Mirande)	2016	Adult	Female	Questing tick, collected on vegetation	0
Amblyomma cajennense	Cayenne tick	Ixodidae	Matoury (Piste de la Mirande)	2016	Adult	Female	Questing tick, collected on vegetation	1
Amblyomma cajennense	Cayenne tick	Ixodidae	Matoury (Piste de la Mirande)	2016	Adult	Female	Questing tick, collected on vegetation	0
Amblyomma cajennense	Cayenne tick	Ixodidae	Matoury (Piste de la Mirande)	2016	Adult	Female	Questing tick, collected on vegetation	0
Amblyomma cajennense	Cayenne tick	Ixodidae	Matoury (Piste de la Mirande)	2016	Adult	Female	Questing tick, collected on vegetation	0
Amblyomma cajennense	Cayenne tick	Ixodidae	Matoury (Piste de la Mirande)	2016	Adult	Female	Questing tick, collected on vegetation	0
Amblyomma cajennense	Cayenne tick	Ixodidae	Matoury (Piste de la Mirande)	2016	Adult	Female	Questing tick, collected on vegetation	0
Amblyomma cajennense	Cayenne tick	Ixodidae	Matoury (Piste de la Mirande)	2016	Adult	Female	Questing tick, collected on vegetation	0
Amblyomma cajennense	Cayenne tick	Ixodidae	Matoury (Piste de la Mirande)	2016	Adult	Female	Questing tick, collected on vegetation	0
Amblyomma cajennense	Cayenne tick	Ixodidae	Matoury (Piste de la Mirande)	2016	Adult	Female	Questing tick, collected on vegetation	0
Amblyomma cajennense	Cayenne tick	Ixodidae	Matoury (Piste de la Mirande)	2016	Adult	Female	Questing tick, collected on vegetation	0
Amblyomma cajennense	Cayenne tick	Ixodidae	Matoury (Piste de la Mirande)	2016	Adult	Female	Questing tick, collected on vegetation	0
Amblyomma cajennense	Cayenne tick	Ixodidae	Matoury (Piste de la Mirande)	2016	Adult	Female	Questing tick, collected on vegetation	0
Amblyomma cajennense	Cayenne tick	Ixodidae	Matoury (Piste de la Mirande)	2016	Adult	Male	Questing tick, collected on vegetation	0
Amblyomma cajennense	Cayenne tick	Ixodidae	Matoury (Piste de la Mirande)	2016	Adult	Male	Questing tick, collected on vegetation	0
Amblyomma cajennense	Cayenne tick	Ixodidae	Matoury (Piste de la Mirande)	2016	Adult	Male	Questing tick, collected on vegetation	0
Amblyomma cajennense	Cayenne tick	Ixodidae	Matoury (Piste de la Mirande)	2016	Adult	Male	Questing tick, collected on vegetation	0
Amblyomma cajennense	Cayenne tick	Ixodidae	Matoury (Piste de la Mirande)	2016	Adult	Male	Questing tick, collected on vegetation	0
Amblyomma cajennense	Cayenne tick	Ixodidae	Matoury (Piste de la Mirande)	2016	Adult	Male	Questing tick, collected on vegetation	1
Amblyomma cajennense	Cayenne tick	Ixodidae	Matoury (Piste de la Mirande)	2016	Adult	Male	Questing tick, collected on vegetation	0
Amblyomma cajennense	Cayenne tick	Ixodidae	Matoury (Piste de la Mirande)	2016	Adult	Male	Questing tick, collected on vegetation	0
Amblyomma cajennense	Cayenne tick	Ixodidae	Matoury (Piste de la Mirande)	2016	Adult	Male	Questing tick, collected on vegetation	0
Amblyomma cajennense	Cayenne tick	Ixodidae	Matoury (Piste de la Mirande)	2016	Adult	Male	Questing tick, collected on vegetation	0
Amblyomma cajennense	Cayenne tick	Ixodidae	Matoury (Piste de la Mirande)	2016	Adult	Male	Questing tick, collected on vegetation	0
Amblyomma cajennense	Cayenne tick	Ixodidae	Matoury (Piste de la Mirande)	2016	Adult	Male	Questing tick, collected on vegetation	0
Amblyomma cajennense	Cayenne tick	Ixodidae	Matoury (Piste de la Mirande)	2016	Adult	Male	Questing tick, collected on vegetation	0
Amblyomma cajennense	Cayenne tick	Ixodidae	Matoury (Piste de la Mirande)	2016	Adult	Male	Questing tick, collected on vegetation	0
Amblyomma cajennense	Cayenne tick	Ixodidae	Matoury (Piste de la Mirande)	2016	Adult	Male	Questing tick, collected on vegetation	0
Amblyomma cajennense	Cayenne tick	Ixodidae	Matoury (Piste de la Mirande)	2016	Adult	Male	Questing tick, collected on vegetation	0
Amblyomma cajennense	Cayenne tick	Ixodidae	Matoury (Piste de la Mirande)	2016	Adult	Male	Questing tick, collected on vegetation	0
Amblyomma cajennense	Cayenne tick	Ixodidae	Matoury (Piste de la Mirande)	2016	Adult	Male	Questing tick, collected on vegetation	0
Amblyomma cajennense	Cayenne tick	Ixodidae	Matoury (Piste de la Mirande)	2016	Adult	Male	Questing tick, collected on vegetation	0
Amblyomma cajennense	Cayenne tick	Ixodidae	Matoury (Piste de la Mirande)	2016	Adult	Male	Questing tick, collected on vegetation	0
Amblyomma cajennense	Cayenne tick	Ixodidae	Matoury (Piste de la Mirande)	2016	Adult	Male	Questing tick, collected on vegetation	0
Amblyomma cajennense	Cayenne tick	Ixodidae	Montagne de Kaw	2016	Nymph	_	Questing tick, collected on vegetation	0
Amblyomma cajennense	Cayenne tick	Ixodidae	Montagne de Kaw	2016	Nymph	_	Questing tick, collected on vegetation	0
Amblyomma cajennense	Cayenne tick	Ixodidae	Montagne de Kaw	2016	Nymph	_	Questing tick, collected on vegetation	0
Amblyomma cajennense	Cayenne tick	Ixodidae	Montagne de Kaw	2016	Nymph	_	Questing tick, collected on vegetation	0
Amblyomma cajennense	Cayenne tick	Ixodidae	Montagne de Kaw	2016	Nymph	_	Questing tick, collected on vegetation	0
Amblyomma cajennense	Cayenne tick	Ixodidae	Montagne de Kaw	2016	Nymph	_	Questing tick, collected on vegetation	0
Amblyomma cajennense	Cayenne tick	Ixodidae	Montagne de Kaw	2016	Nymph	_	Questing tick, collected on vegetation	0
Amblyomma cajennense	Cayenne tick	Ixodidae	Montagne de Kaw	2016	Nymph	_	Questing tick, collected on vegetation	0
Amblyomma cajennense	Cayenne tick	Ixodidae	Montagne de Kaw	2016	Nymph	_	Questing tick, collected on vegetation	0
Amblyomma cajennense	Cayenne tick	Ixodidae	Montagne de Kaw	2016	Nymph	_	Questing tick, collected on vegetation	0
Amblyomma cajennense	Cayenne tick	Ixodidae	Montagne de Kaw	2016	Nymph	_	Questing tick, collected on vegetation	0
Amblyomma cajennense	Cayenne tick	Ixodidae	Montagne de Kaw	2016	Nymph	_	Questing tick, collected on vegetation	0
Amblyomma cajennense	Cayenne tick	Ixodidae	Montagne de Kaw	2016	Nymph	_	Questing tick, collected on vegetation	0
Amblyomma cajennense	Cayenne tick	Ixodidae	Montagne de Kaw	2016	Nymph	_	Questing tick, collected on vegetation	0
Amblyomma cajennense	Cayenne tick	Ixodidae	Montagne de Kaw	2016	Nymph	_	Questing tick, collected on vegetation	0
Amblyomma cajennense	Cayenne tick	Ixodidae	Montagne de Kaw	2016	Nymph	_	Questing tick, collected on vegetation	0
Amblyomma cajennense	Cayenne tick	Ixodidae	Montagne de Kaw	2016	Nymph	_	Questing tick, collected on vegetation	0
Amblyomma cajennense	Cayenne tick	Ixodidae	Montagne de Kaw	2016	Nymph	_	Questing tick, collected on vegetation	0
Amblyomma cajennense	Cayenne tick	Ixodidae	Montagne de Kaw	2016	Nymph	_	Questing tick, collected on vegetation	0
Amblyomma cajennense	Cayenne tick	Ixodidae	Montagne de Kaw	2016	Nymph	_	Questing tick, collected on vegetation	0
Amblyomma cajennense	Cayenne tick	Ixodidae	Montagne de Kaw	2016	Nymph	_	Questing tick, collected on vegetation	0
Amblyomma cajennense	Cayenne tick	Ixodidae	Montagne de Kaw	2016	Nymph	_	Questing tick, collected on vegetation	0
Amblyomma cajennense	Cayenne tick	Ixodidae	Montagne de Kaw	2016	Nymph	_	Questing tick, collected on vegetation	0
Amblyomma cajennense	Cayenne tick	Ixodidae	Montagne de Kaw	2016	Nymph	_	Questing tick, collected on vegetation	0
Amblyomma cajennense	Cayenne tick	Ixodidae	Montagne de Kaw	2016	Nymph	_	Questing tick, collected on vegetation	0
Amblyomma cajennense	Cayenne tick	Ixodidae	Montagne de Kaw	2016	Nymph	_	Questing tick, collected on vegetation	0
Amblyomma cajennense	Cayenne tick	Ixodidae	Montagne de Kaw	2016	Nymph	_	Questing tick, collected on vegetation	0
Amblyomma cajennense	Cayenne tick	Ixodidae	Montagne de Kaw	2016	Nymph	_	Questing tick, collected on vegetation	0
Amblyomma cajennense	Cayenne tick	Ixodidae	Matoury (Piste de la Mirande)	2018	Adult	Female	Questing tick, collected on vegetation	0
Amblyomma cajennense	Cayenne tick	Ixodidae	Matoury (Piste de la Mirande)	2018	Adult	Female	Questing tick, collected on vegetation	0
Amblyomma cajennense	Cayenne tick	Ixodidae	Matoury (Piste de la Mirande)	2018	Adult	Female	Questing tick, collected on vegetation	0
Amblyomma cajennense	Cayenne tick	Ixodidae	Matoury (Piste de la Mirande)	2018	Adult	Female	Questing tick, collected on vegetation	0
Amblyomma cajennense	Cayenne tick	Ixodidae	Matoury (Piste de la Mirande)	2018	Adult	Female	Questing tick, collected on vegetation	0
Amblyomma cajennense	Cayenne tick	Ixodidae	Sinnamary (Piste Saint-Elie)	2016	Nymph	_	Questing tick, collected on vegetation	0
Amblyomma cajennense	Cayenne tick	Ixodidae	Sinnamary (Piste Saint-Elie)	2016	Nymph	_	Questing tick, collected on vegetation	0
Amblyomma cajennense	Cayenne tick	Ixodidae	Sinnamary (Piste Saint-Elie)	2016	Nymph	_	Questing tick, collected on vegetation	0
Amblyomma cajennense	Cayenne tick	Ixodidae	Sinnamary (Piste Saint-Elie)	2016	Nymph	_	Questing tick, collected on vegetation	0
Amblyomma cajennense	Cayenne tick	Ixodidae	Sinnamary (Piste Saint-Elie)	2016	Nymph	_	Questing tick, collected on vegetation	0
Amblyomma cajennense	Cayenne tick	Ixodidae	Sinnamary (Piste Saint-Elie)	2016	Nymph	_	Questing tick, collected on vegetation	0
Amblyomma cajennense	Cayenne tick	Ixodidae	Sinnamary (Piste Saint-Elie)	2016	Nymph	_	Questing tick, collected on vegetation	0
Amblyomma cajennense	Cayenne tick	Ixodidae	Sinnamary (Piste Saint-Elie)	2016	Nymph	_	Questing tick, collected on vegetation	0
Amblyomma cajennense	Cayenne tick	Ixodidae	Sinnamary (Piste Saint-Elie)	2016	Nymph	_	Questing tick, collected on vegetation	0
Amblyomma cajennense	Cayenne tick	Ixodidae	Sinnamary (Piste Saint-Elie)	2016	Nymph	_	Questing tick, collected on vegetation	0
Amblyomma cajennense	Cayenne tick	Ixodidae	Sinnamary (Piste Saint-Elie)	2016	Nymph	_	Questing tick, collected on vegetation	0
Amblyomma cajennense	Cayenne tick	Ixodidae	Sinnamary (Piste Saint-Elie)	2016	Nymph	_	Questing tick, collected on vegetation	0
Amblyomma cajennense	Cayenne tick	Ixodidae	Sinnamary (Piste Saint-Elie)	2016	Nymph	_	Questing tick, collected on vegetation	0
Amblyomma cajennense	Cayenne tick	Ixodidae	Sinnamary (Piste Saint-Elie)	2016	Nymph	_	Questing tick, collected on vegetation	0
Amblyomma cajennense	Cayenne tick	Ixodidae	Sinnamary (Piste Saint-Elie)	2016	Nymph	_	Questing tick, collected on vegetation	0
Amblyomma cajennense	Cayenne tick	Ixodidae	Sinnamary (Piste Saint-Elie)	2016	Nymph	_	Questing tick, collected on vegetation	0
Amblyomma cajennense	Cayenne tick	Ixodidae	Sinnamary (Piste Saint-Elie)	2016	Nymph	_	Questing tick, collected on vegetation	0
Amblyomma cajennense	Cayenne tick	Ixodidae	Sinnamary (Piste Saint-Elie)	2016	Nymph	_	Questing tick, collected on vegetation	0
Amblyomma cajennense	Cayenne tick	Ixodidae	Sinnamary (Piste Saint-Elie)	2016	Nymph	_	Questing tick, collected on vegetation	0
Amblyomma cajennense	Cayenne tick	Ixodidae	Sinnamary (Piste Saint-Elie)	2016	Nymph	_	Questing tick, collected on vegetation	0
Amblyomma cajennense	Cayenne tick	Ixodidae	Sinnamary (Piste Saint-Elie)	2016	Nymph	_	Questing tick, collected on vegetation	0
Amblyomma cajennense	Cayenne tick	Ixodidae	Sinnamary (Piste Saint-Elie)	2016	Nymph	_	Questing tick, collected on vegetation	0
Amblyomma cajennense	Cayenne tick	Ixodidae	Sinnamary (Piste Saint-Elie)	2016	Nymph	_	Questing tick, collected on vegetation	0
Amblyomma cajennense	Cayenne tick	Ixodidae	Sinnamary (Piste Saint-Elie)	2016	Nymph	_	Questing tick, collected on vegetation	0
Amblyomma cajennense	Cayenne tick	Ixodidae	Sinnamary (Piste Saint-Elie)	2016	Nymph	_	Questing tick, collected on vegetation	0
Amblyomma cajennense	Cayenne tick	Ixodidae	Sinnamary (Piste Saint-Elie)	2016	Nymph	_	Questing tick, collected on vegetation	0
Amblyomma cajennense	Cayenne tick	Ixodidae	Sinnamary (Piste Saint-Elie)	2016	Nymph	_	Questing tick, collected on vegetation	0
Amblyomma cajennense	Cayenne tick	Ixodidae	Sinnamary (Piste Saint-Elie)	2016	Nymph	_	Questing tick, collected on vegetation	0
Amblyomma cajennense	Cayenne tick	Ixodidae	Sinnamary (Piste Saint-Elie)	2016	Nymph	_	Questing tick, collected on vegetation	0
Amblyomma cajennense	Cayenne tick	Ixodidae	Kourou (Montagne des Singes)	2016	Nymph	_	Questing tick, collected on vegetation	0
Amblyomma cajennense	Cayenne tick	Ixodidae	Kourou (Montagne des Singes)	2016	Nymph	_	Questing tick, collected on vegetation	0
Amblyomma cajennense	Cayenne tick	Ixodidae	Kourou (Montagne des Singes)	2016	Nymph	_	Questing tick, collected on vegetation	0
Amblyomma cajennense	Cayenne tick	Ixodidae	Kourou (Montagne des Singes)	2016	Nymph	_	Questing tick, collected on vegetation	0
Amblyomma cajennense	Cayenne tick	Ixodidae	Kourou (Montagne des Singes)	2016	Nymph	_	Questing tick, collected on vegetation	0
Amblyomma cajennense	Cayenne tick	Ixodidae	Kourou (Montagne des Singes)	2016	Nymph	_	Questing tick, collected on vegetation	0
Amblyomma cajennense	Cayenne tick	Ixodidae	Kourou (Montagne des Singes)	2016	Nymph	_	Questing tick, collected on vegetation	0
Amblyomma cajennense	Cayenne tick	Ixodidae	Kourou (Montagne des Singes)	2016	Nymph	_	Questing tick, collected on vegetation	0
Amblyomma calcaratum	_	Ixodidae	Kourou (Montagne des Singes)	2016	Larva	_	Questing tick, collected on vegetation	0
Amblyomma coelebs	_	Ixodidae	Sinnamary (Crique Verte)	2016	Nymph	_	Questing tick, collected on human	0
Amblyomma coelebs	_	Ixodidae	Montagne de Kaw	2016	Nymph	_	Questing tick, collected on vegetation	0
Amblyomma coelebs	_	Ixodidae	Montagne de Kaw	2016	Nymph	_	Questing tick, collected on vegetation	0
Amblyomma coelebs	_	Ixodidae	Petit-Saut (Montagne Plomb)	2016	Nymph	_	Questing tick, collected on vegetation	0
Amblyomma coelebs	_	Ixodidae	Petit-Saut (Montagne Plomb)	2016	Nymph	_	Questing tick, collected on vegetation	0
Amblyomma coelebs	_	Ixodidae	Petit-Saut (Montagne Plomb)	2016	Nymph	_	Questing tick, collected on vegetation	0
Amblyomma coelebs	_	Ixodidae	Petit-Saut (Montagne Plomb)	2016	Nymph	_	Questing tick, collected on vegetation	0
Amblyomma coelebs	_	Ixodidae	Petit-Saut (Montagne Plomb)	2016	Nymph	_	Questing tick, collected on vegetation	0
Amblyomma coelebs	_	Ixodidae	Petit-Saut (Montagne Plomb)	2016	Nymph	_	Questing tick, collected on vegetation	0
Amblyomma coelebs	_	Ixodidae	Petit-Saut (Montagne Plomb)	2016	Nymph	_	Questing tick, collected on vegetation	0
Amblyomma coelebs	_	Ixodidae	Petit-Saut (Montagne Plomb)	2016	Nymph	_	Questing tick, collected on vegetation	0
Amblyomma coelebs	_	Ixodidae	Petit-Saut (Montagne Plomb)	2016	Nymph	_	Questing tick, collected on vegetation	0
Amblyomma coelebs	_	Ixodidae	Petit-Saut (Montagne Plomb)	2016	Nymph	_	Questing tick, collected on vegetation	0
Amblyomma coelebs	_	Ixodidae	Route de Kaw	2017	Nymph	_	Questing tick, collected on human	0
Amblyomma coelebs	_	Ixodidae	Kourou (CSG)	2016	Nymph	_	Questing tick, collected on human	0
Amblyomma coelebs	_	Ixodidae	Kourou (CSG)	2016	Nymph	_	Questing tick, collected on human	0
Amblyomma coelebs	_	Ixodidae	Kourou (CSG)	2016	Nymph	_	Questing tick, collected on human	0
Amblyomma coelebs	_	Ixodidae	Nouragues	2016	Nymph	_	Questing tick, collected on human	0

Amblyomma coelebs	_	Ixodidae	Nouragues	2016	Nymph	_	Questing tick, collected on human	0
Amblyomma coelebs	_	Ixodidae	St-Georges de l'Oyapock (Crique Gabaret)	2016	Nymph	_	Questing tick, collected on human	0
Amblyomma coelebs	_	Ixodidae	St-Georges de l'Oyapock (Crique Gabaret)	2016	Nymph	_	Questing tick, collected on human	0
Amblyomma coelebs	_	Ixodidae	Petit-Saut (Montagne Plomb)	2016	Nymph	_	Questing tick, collected on vegetation	0
Amblyomma coelebs	_	Ixodidae	Petit-Saut (Montagne Plomb)	2016	Nymph	_	Questing tick, collected on vegetation	0
Amblyomma coelebs	_	Ixodidae	Petit-Saut (Montagne Plomb)	2016	Nymph	_	Questing tick, collected on vegetation	0
Amblyomma coelebs	_	Ixodidae	Petit-Saut (Montagne Plomb)	2016	Nymph	_	Questing tick, collected on vegetation	0
Amblyomma coelebs	_	Ixodidae	Petit-Saut (Montagne Plomb)	2016	Nymph	_	Questing tick, collected on vegetation	0
Amblyomma coelebs	_	Ixodidae	Petit-Saut (Montagne Plomb)	2016	Nymph	_	Questing tick, collected on vegetation	0
Amblyomma coelebs	_	Ixodidae	Apatou (Crique Grande Absinthe)	2016	Nymph	_	Questing tick, collected on vegetation	0
Amblyomma coelebs	_	Ixodidae	Apatou (Crique Grande Absinthe)	2016	Nymph	_	Questing tick, collected on vegetation	0
Amblyomma coelebs	_	Ixodidae	Mana (Trinité)	2017	Nymph	_	Questing tick, collected on human	0
Amblyomma coelebs	_	Ixodidae	Petit-Saut (Carbet Hydreco)	2017	Nymph	_	Questing tick, collected on human	0
Amblyomma dissimile	Iguana tick	Ixodidae	Talhuen (Village)	2016	Adult	Female	Questing tick, collected on vegetation	0
Amblyomma dissimile	Iguana tick	Ixodidae	Talhuen (Village)	2016	Adult	Female	Questing tick, collected on vegetation	0
Amblyomma dissimile	Iguana tick	Ixodidae	Awala-Yalimapo (Ayawande)	2016	Adult	Male	Engorged tick, collected on cane toad	0
Amblyomma dissimile	Iguana tick	Ixodidae	Awala-Yalimapo (Ayawande)	2016	Adult	Male	Engorged tick, collected on cane toad	0
Amblyomma dissimile	Iguana tick	Ixodidae	Awala-Yalimapo (Ayawande)	2016	Nymph	_	Engorged tick, collected on cane toad	0
Amblyomma dissimile	Iguana tick	Ixodidae	Awala-Yalimapo (Ayawande)	2016	Nymph	_	Engorged tick, collected on cane toad	0
Amblyomma dissimile	Iguana tick	Ixodidae	Rémire-Montjoly	2018	Adult	Female	Engorged tick, collected on green iguana	0
Amblyomma dissimile	Iguana tick	Ixodidae	Rémire-Montjoly	2018	Adult	Female	Engorged tick, collected on green iguana	0
Amblyomma dissimile	Iguana tick	Ixodidae	Rémire-Montjoly	2018	Adult	Female	Engorged tick, collected on green iguana	0
Amblyomma dissimile	Iguana tick	Ixodidae	Rémire-Montjoly	2017	Adult	Female	Engorged tick, collected on green iguana	0
Amblyomma dissimile	Iguana tick	Ixodidae	Rémire-Montjoly	2017	Adult	Female	Engorged tick, collected on green iguana	0
Amblyomma dissimile	Iguana tick	Ixodidae	Rémire-Montjoly	2017	Adult	Male	Engorged tick, collected on green iguana	0
Amblyomma dissimile	Iguana tick	Ixodidae	Rémire-Montjoly	2017	Adult	Male	Engorged tick, collected on green iguana	0
Amblyomma dissimile	Iguana tick	Ixodidae	Rémire-Montjoly	2017	Adult	Male	Engorged tick, collected on green iguana	0
Amblyomma dissimile	Iguana tick	Ixodidae	Rémire-Montjoly	2017	Adult	Male	Engorged tick, collected on green iguana	0
Amblyomma dissimile	Iguana tick	Ixodidae	Rémire-Montjoly	2017	Adult	Male	Engorged tick, collected on green iguana	0
Amblyomma dissimile	Iguana tick	Ixodidae	Rémire-Montjoly	2017	Adult	Male	Engorged tick, collected on green iguana	0
Amblyomma dissimile	Iguana tick	Ixodidae	Rémire-Montjoly	2017	Adult	Male	Engorged tick, collected on green iguana	0
Amblyomma dissimile	Iguana tick	Ixodidae	Rémire-Montjoly	2017	Adult	Male	Engorged tick, collected on green iguana	0
Amblyomma dissimile	Iguana tick	Ixodidae	Rémire-Montjoly	2017	Adult	Male	Engorged tick, collected on green iguana	0
Amblyomma dissimile	Iguana tick	Ixodidae	Rémire-Montjoly	2017	Adult	Male	Engorged tick, collected on green iguana	0
Amblyomma geayi	_	Ixodidae	Rémire-Montjoly (Vidal)	2016	Adult	Male	Engorged tick, collected on sloth	0
Amblyomma geayi	_	Ixodidae	Rémire-Montjoly (Vidal)	2016	Adult	Male	Engorged tick, collected on sloth	0
Amblyomma geayi	_	Ixodidae	Cayenne (Montagne du Tigre)	2017	Larva	_	Questing tick, collected on vegetation	0
Amblyomma geayi	_	Ixodidae	Cayenne (Montagne du Tigre)	2017	Larva	_	Questing tick, collected on vegetation	0
Amblyomma geayi	_	Ixodidae	Cayenne (Montagne du Tigre)	2017	Larva	_	Questing tick, collected on vegetation	0
Amblyomma geayi	_	Ixodidae	Cayenne (Montagne du Tigre)	2017	Larva	_	Questing tick, collected on vegetation	0
Amblyomma geayi	_	Ixodidae	Rémire-Montjoly (Sentier du Rorota)	2014	Larva	_	Engorged tick, collected on passerine	0
Amblyomma geayi	_	Ixodidae	Rémire-Montjoly (Sentier du Rorota)	2014	Larva	_	Engorged tick, collected on passerine	0
Amblyomma geayi	_	Ixodidae	Rémire-Montjoly (Sentier du Rorota)	2014	Larva	_	Engorged tick, collected on passerine	0
Amblyomma geayi	_	Ixodidae	Matoury (Mont Paramana)	2014	Larva	_	Engorged tick, collected on passerine	0
Amblyomma goeldii	_	Ixodidae	Régina (N1 road PK63)	2017	Adult	Female	Engorged tick, collected on tamandua	0
Amblyomma goeldii	_	Ixodidae	Régina (N1 road PK63)	2017	Adult	Female	Engorged tick, collected on tamandua	0
Amblyomma goeldii	_	Ixodidae	Régina (N1 road PK63)	2017	Adult	Female	Engorged tick, collected on tamandua	0
Amblyomma goeldii	_	Ixodidae	Régina (N1 road PK63)	2017	Adult	Male	Engorged tick, collected on tamandua	0
Amblyomma goeldii	_	Ixodidae	Régina (N1 road PK63)	2017	Adult	Male	Engorged tick, collected on tamandua	0
Amblyomma humerale	_	Ixodidae	Kourou (Montagne des Singes)	2016	Nymph	_	Questing tick, collected on human	0
Amblyomma humerale	_	Ixodidae	Matoury (Route nationale)	2016	Nymph	_	Engorged tick, collected on opossum	0
Amblyomma humerale	_	Ixodidae	Matoury (Route nationale)	2016	Nymph	_	Engorged tick, collected on opossum	0
Amblyomma humerale	_	Ixodidae	Mana (Trinité)	2017	Adult	Female	Questing tick, collected on human	0
Amblyomma humerale	_	Ixodidae	Nouragues	2017	Adult	Male	Questing tick, collected on vegetation	0
Amblyomma humerale	_	Ixodidae	Nouragues	2017	Adult	Male	Questing tick, collected on vegetation	0
Amblyomma humerale	_	Ixodidae	Nouragues	2017	Adult	Male	Questing tick, collected on vegetation	0
Amblyomma humerale	_	Ixodidae	Nouragues	2017	Adult	Male	Questing tick, collected on vegetation	0
Amblyomma humerale	_	Ixodidae	Nouragues	2017	Adult	Male	Questing tick, collected on vegetation	0
Amblyomma humerale	_	Ixodidae	Kourou (Montagne des Pères)	2014	Nymph	_	Questing tick, collected on passerine	0
Amblyomma latepunctatum	_	Ixodidae	Petit-Saut (Montagne Plomb)	2016	Nymph	_	Questing tick, collected on vegetation	0
Amblyomma latepunctatum	_	Ixodidae	Petit-Saut (Montagne Plomb)	2016	Nymph	_	Questing tick, collected on vegetation	0
Amblyomma latepunctatum	_	Ixodidae	Petit-Saut (Montagne Plomb)	2016	Nymph	_	Questing tick, collected on vegetation	0
Amblyomma latepunctatum	_	Ixodidae	St-Georges de l'Oyapock (Crique Gabaret)	2016	Nymph	_	Questing tick, collected on vegetation	0
Amblyomma longirostre	_	Ixodidae	Rémire-Montjoly (Sentier du Rorota)	2016	Larva	_	Engorged tick, collected on passerine	0
Amblyomma longirostre	_	Ixodidae	Rémire-Montjoly (Sentier du Rorota)	2016	Larva	_	Engorged tick, collected on passerine	0
Amblyomma longirostre	_	Ixodidae	Rémire-Montjoly (Sentier du Rorota)	2016	Larva	_	Engorged tick, collected on passerine	0
Amblyomma longirostre	_	Ixodidae	Rémire-Montjoly (Sentier du Rorota)	2016	Larva	_	Engorged tick, collected on passerine	0
Amblyomma longirostre	_	Ixodidae	Rémire-Montjoly (Sentier du Rorota)	2016	Larva	_	Engorged tick, collected on passerine	0
Amblyomma longirostre	_	Ixodidae	Rémire-Montjoly (Sentier du Rorota)	2016	Larva	_	Engorged tick, collected on passerine	0
Amblyomma longirostre	_	Ixodidae	Rémire-Montjoly (Sentier du Rorota)	2016	Larva	_	Engorged tick, collected on passerine	0
Amblyomma longirostre	_	Ixodidae	Rémire-Montjoly (Sentier du Rorota)	2016	Larva	_	Engorged tick, collected on passerine	0
Amblyomma longirostre	_	Ixodidae	Rémire-Montjoly (Sentier du Rorota)	2016	Larva	_	Engorged tick, collected on passerine	0
Amblyomma longirostre	_	Ixodidae	Rémire-Montjoly (Sentier du Rorota)	2016	Larva	_	Engorged tick, collected on passerine	0
Amblyomma longirostre	_	Ixodidae	Rémire-Montjoly (Sentier du Rorota)	2016	Larva	_	Engorged tick, collected on passerine	0
Amblyomma longirostre	_	Ixodidae	Rémire-Montjoly (Sentier du Rorota)	2016	Larva	_	Engorged tick, collected on passerine	0
Amblyomma longirostre	_	Ixodidae	Rémire-Montjoly (Sentier du Rorota)	2016	Larva	_	Engorged tick, collected on passerine	0
Amblyomma longirostre	_	Ixodidae	Rémire-Montjoly (Sentier du Rorota)	2016	Larva	_	Engorged tick, collected on passerine	0
Amblyomma longirostre	_	Ixodidae	Rémire-Montjoly (Sentier du Rorota)	2016	Larva	_	Engorged tick, collected on passerine	0
Amblyomma longirostre	_	Ixodidae	Rémire-Montjoly (Sentier du Rorota)	2016	Larva	_	Engorged tick, collected on passerine	0
Amblyomma longirostre	_	Ixodidae	Rémire-Montjoly (Sentier du Rorota)	2016	Larva	_	Engorged tick, collected on passerine	0
Amblyomma longirostre	_	Ixodidae	Rémire-Montjoly (Sentier du Rorota)	2016	Larva	_	Engorged tick, collected on passerine	0
Amblyomma longirostre	_	Ixodidae	Rémire-Montjoly (Sentier du Rorota)	2016	Larva	_	Engorged tick, collected on passerine	0
Amblyomma longirostre	_	Ixodidae	Rémire-Montjoly (Sentier du Rorota)	2016	Larva	_	Engorged tick, collected on passerine	0
Amblyomma longirostre	_	Ixodidae	Rémire-Montjoly (Sentier du Rorota)	2016	Larva	_	Engorged tick, collected on passerine	0
Amblyomma longirostre	_	Ixodidae	Rémire-Montjoly (Sentier du Rorota)	2016	Larva	_	Engorged tick, collected on passerine	0
Amblyomma longirostre	_	Ixodidae	Rémire-Montjoly (Sentier du Rorota)	2016	Larva	_	Engorged tick, collected on passerine	0
Amblyomma longirostre	_	Ixodidae	Rémire-Montjoly (Sentier du Rorota)	2014	Larva	_	Engorged tick, collected on passerine	0
Amblyomma longirostre	_	Ixodidae	Rémire-Montjoly (Sentier du Rorota)	2014	Larva	_	Engorged tick, collected on passerine	0
Amblyomma longirostre	_	Ixodidae	Rémire-Montjoly (Sentier du Rorota)	2014	Larva	_	Engorged tick, collected on passerine	0
Amblyomma longirostre	_	Ixodidae	Rémire-Montjoly (Sentier du Rorota)	2014	Larva	_	Engorged tick, collected on passerine	0
Amblyomma longirostre	_	Ixodidae	Rémire-Montjoly (Sentier du Rorota)	2014	Larva	_	Engorged tick, collected on passerine	0
Amblyomma longirostre	_	Ixodidae	Rémire-Montjoly (Sentier du Rorota)	2014	Larva	_	Engorged tick, collected on passerine	0
Amblyomma longirostre	_	Ixodidae	Rémire-Montjoly (Sentier du Rorota)	2014	Larva	_	Engorged tick, collected on passerine	0
Amblyomma longirostre	_	Ixodidae	Rémire-Montjoly (Sentier du Rorota)	2014	Larva	_	Engorged tick, collected on passerine	0
Amblyomma longirostre	_	Ixodidae	Rémire-Montjoly (Sentier du Rorota)	2014	Larva	_	Engorged tick, collected on passerine	0
Amblyomma longirostre	_	Ixodidae	Rémire-Montjoly (Sentier du Rorota)	2014	Larva	_	Engorged tick, collected on passerine	0
Amblyomma longirostre	_	Ixodidae	Rémire-Montjoly (Sentier du Rorota)	2014	Larva	_	Engorged tick, collected on passerine	0
Amblyomma longirostre	_	Ixodidae	Rémire-Montjoly (Sentier du Rorota)	2014	Larva	_	Engorged tick, collected on passerine	0
Amblyomma longirostre	_	Ixodidae	Rémire-Montjoly (Sentier du Rorota)	2014	Larva	_	Engorged tick, collected on passerine	0
Amblyomma longirostre	_	Ixodidae	Rémire-Montjoly (Sentier du Rorota)	2014	Larva	_	Engorged tick, collected on passerine	0
Amblyomma longirostre	_	Ixodidae	Rémire-Montjoly (Sentier du Rorota)	2014	Larva	_	Engorged tick, collected on passerine	0
Amblyomma longirostre	_	Ixodidae	Rémire-Montjoly (Sentier du Rorota)	2014	Larva	_	Engorged tick, collected on passerine	0
Amblyomma longirostre	_	Ixodidae	Rémire-Montjoly (Sentier du Rorota)	2014	Larva	_	Engorged tick, collected on passerine	0
Amblyomma longirostre	_	Ixodidae	Rémire-Montjoly (Sentier du Rorota)	2014	Larva	_	Engorged tick, collected on passerine	0
Amblyomma longirostre	_	Ixodidae	Rémire-Montjoly (Sentier du Rorota)	2014	Larva	_	Engorged tick, collected on passerine	0
Amblyomma longirostre	_	Ixodidae	Rémire-Montjoly (Sentier du Rorota)	2014	Larva	_	Engorged tick, collected on passerine	0
Amblyomma longirostre	_	Ixodidae	Rémire-Montjoly (Sentier du Rorota)	2014	Larva	_	Engorged tick, collected on passerine	0
Amblyomma longirostre	_	Ixodidae	Rémire-Montjoly (Sentier du Rorota)	2014	Larva	_	Engorged tick, collected on passerine	0
Amblyomma longirostre	_	Ixodidae	Rémire-Montjoly (Sentier du Rorota)	2014	Larva	_	Engorged tick, collected on passerine	0
Amblyomma longirostre	_	Ixodidae	Rémire-Montjoly (Sentier du Rorota)	2014	Larva	_	Engorged tick, collected on passerine	0
Amblyomma longirostre	_	Ixodidae	Rémire-Montjoly (Sentier du Rorota)	2014	Larva	_	Engorged tick, collected on passerine	0
Amblyomma longirostre	_	Ixodidae	Rémire-Montjoly (Sentier du Rorota)	2014	Larva	_	Engorged tick, collected on passerine	0
Amblyomma longirostre	_	Ixodidae	Rémire-Montjoly (Sentier du Rorota)	2014	Larva	_	Engorged tick, collected on passerine	0
Amblyomma longirostre	_	Ixodidae	Rémire-Montjoly (Sentier du Rorota)	2014	Larva	_	Engorged tick, collected on passerine	0
Amblyomma longirostre	_	Ixodidae	Rémire-Montjoly (Sentier du Rorota)	2014	Larva	_	Engorged tick, collected on passerine	0
Amblyomma longirostre	_	Ixodidae	Rémire-Montjoly (Sentier du Rorota)	2014	Larva	_	Engorged tick, collected on passerine	0
Amblyomma longirostre	_	Ixodidae	Rémire-Montjoly (Sentier du Rorota)	2014	Larva	_	Engorged tick, collected on passerine	0
Amblyomma longirostre	_	Ixodidae	Rémire-Montjoly (Sentier du Rorota)	2014	Larva	_	Engorged tick, collected on passerine	0
Amblyomma longirostre	_	Ixodidae	Rémire-Montjoly (Sentier du Rorota)	2014	Larva	_	Engorged tick, collected on passerine	0
Amblyomma longirostre	_	Ixodidae	Saint-Laurent-du-Maroni (Saint-Jean)	2013	Larva	_	Engorged tick, collected on passerine	0
Amblyomma longirostre	_	Ixodidae	Saint-Laurent-du-Maroni (Saint-Jean)	2013	Larva	_	Engorged tick, collected on passerine	0
Amblyomma longirostre	_	Ixodidae	Saint-Laurent-du-Maroni (Piste Voltaire)	2014	Larva	_	Engorged tick, collected on passerine	0
Amblyomma longirostre	_	Ixodidae	Kourou (Montagne des Singes)	2014	Larva	_	Engorged tick, collected on passerine	0
Amblyomma longirostre	_	Ixodidae	Kourou (Montagne des Singes)	2014	Nymph	_	Engorged tick, collected on passerine	0
Amblyomma longirostre	_	Ixodidae	Kourou (Montagne des Singes)	2014	Larva	_	Engorged tick, collected on passerine	0
Amblyomma longirostre	_	Ixodidae	Kourou (Montagne des Singes)	2014	Larva	_	Engorged tick, collected on passerine	0
Amblyomma longirostre	_	Ixodidae	Kourou (Montagne des Singes)	2014	Larva	_	Engorged tick, collected on passerine	0
Amblyomma longirostre	_	Ixodidae	Montsinéry (Bagne des Annamites)	2014	Larva	_	Engorged tick, collected on passerine	0
Amblyomma longirostre	_	Ixodidae	Montsinéry (Bagne des Annamites)	2014	Larva	_	Engorged tick, collected on passerine	0
Amblyomma longirostre	_	Ixodidae	Kourou (Montagne des Pères)	2014	Larva	_	Engorged tick, collected on passerine	0
Amblyomma longirostre	_	Ixodidae	Kourou (Montagne des Pères)	2014	Larva	_	Engorged tick, collected on passerine	0
Amblyomma longirostre	_	Ixodidae	Kourou (Montagne des Pères)	2014	Larva	_	Engorged tick, collected on passerine	0
Amblyomma longirostre	_	Ixodidae	Kourou (Montagne des Pères)	2014	Larva	_	Engorged tick, collected on passerine	0
Amblyomma longirostre	_	Ixodidae	Kourou (Montagne des Pères)	2014	Larva	_	Engorged tick, collected on passerine	0

Amblyomma longirostre	_	Ixodidae	Kourou (Montagne des Pères)	2014	Larva	_	Engorged tick, collected on passerine	0
Amblyomma longirostre	_	Ixodidae	Kourou (Montagne des Pères)	2014	Larva	_	Engorged tick, collected on passerine	0
Amblyomma longirostre	_	Ixodidae	Kourou (Montagne des Pères)	2014	Larva	_	Engorged tick, collected on passerine	0
Amblyomma longirostre	_	Ixodidae	Kourou (Montagne des Pères)	2014	Larva	_	Engorged tick, collected on passerine	0
Amblyomma longirostre	_	Ixodidae	Kourou (Montagne des Pères)	2014	Larva	_	Engorged tick, collected on passerine	0
Amblyomma longirostre	_	Ixodidae	Kourou (Montagne des Pères)	2014	Larva	_	Engorged tick, collected on passerine	0
Amblyomma longirostre	_	Ixodidae	Macouria (Lotissement Maillard)	2012	Larva	_	Engorged tick, collected on passerine	0
Amblyomma longirostre	_	Ixodidae	Macouria (Lotissement Maillard)	2012	Larva	_	Engorged tick, collected on passerine	0
Amblyomma longirostre	_	Ixodidae	Macouria (Lotissement Maillard)	2012	Larva	_	Engorged tick, collected on passerine	0
Amblyomma longirostre	_	Ixodidae	Macouria (Lotissement Maillard)	2012	Larva	_	Engorged tick, collected on passerine	0
Amblyomma longirostre	_	Ixodidae	Macouria (Lotissement Maillard)	2012	Larva	_	Engorged tick, collected on passerine	0
Amblyomma longirostre	_	Ixodidae	Macouria (Lotissement Maillard)	2012	Larva	_	Engorged tick, collected on passerine	0
Amblyomma longirostre	_	Ixodidae	Macouria (Lotissement Maillard)	2012	Larva	_	Engorged tick, collected on passerine	0
Amblyomma longirostre	_	Ixodidae	Macouria (Lotissement Maillard)	2012	Larva	_	Engorged tick, collected on passerine	0
Amblyomma longirostre	_	Ixodidae	Macouria (Lotissement Maillard)	2012	Larva	_	Engorged tick, collected on passerine	0
Amblyomma longirostre	_	Ixodidae	Macouria (Lotissement Maillard)	2012	Larva	_	Engorged tick, collected on passerine	0
Amblyomma longirostre	_	Ixodidae	Macouria (Lotissement Maillard)	2012	Larva	_	Engorged tick, collected on passerine	0
Amblyomma longirostre	_	Ixodidae	Macouria (Lotissement Maillard)	2012	Larva	_	Engorged tick, collected on passerine	0
Amblyomma longirostre	_	Ixodidae	Macouria (Lotissement Maillard)	2012	Larva	_	Engorged tick, collected on passerine	0
Amblyomma longirostre	_	Ixodidae	Macouria (Lotissement Maillard)	2012	Larva	_	Engorged tick, collected on passerine	0
Amblyomma longirostre	_	Ixodidae	Macouria (Lotissement Maillard)	2012	Larva	_	Engorged tick, collected on passerine	0
Amblyomma longirostre	_	Ixodidae	Macouria (Lotissement Maillard)	2012	Larva	_	Engorged tick, collected on passerine	0
Amblyomma longirostre	_	Ixodidae	Macouria (Lotissement Maillard)	2012	Larva	_	Engorged tick, collected on passerine	0
Amblyomma longirostre	_	Ixodidae	Macouria (Lotissement Maillard)	2012	Larva	_	Engorged tick, collected on passerine	0
Amblyomma longirostre	_	Ixodidae	Matoury (PK7)	2012	Larva	_	Engorged tick, collected on passerine	0
Amblyomma longirostre	_	Ixodidae	Macouria (Lotissement Maillard)	2013	Larva	_	Engorged tick, collected on passerine	0
Amblyomma longirostre	_	Ixodidae	Macouria (Lotissement Maillard)	2013	Larva	_	Engorged tick, collected on passerine	0
Amblyomma longirostre	_	Ixodidae	Macouria (Lotissement Maillard)	2013	Larva	_	Engorged tick, collected on passerine	0
Amblyomma longirostre	_	Ixodidae	Macouria (Lotissement Maillard)	2014	Larva	_	Engorged tick, collected on passerine	0
Amblyomma longirostre	_	Ixodidae	Macouria (Lotissement Maillard)	2014	Nymph	_	Engorged tick, collected on passerine	0
Amblyomma longirostre	_	Ixodidae	Macouria (Lotissement Maillard)	2014	Larva	_	Engorged tick, collected on passerine	0
Amblyomma longirostre	_	Ixodidae	Macouria (Lotissement Maillard)	2014	Larva	_	Engorged tick, collected on passerine	0
Amblyomma longirostre	_	Ixodidae	Macouria (Lotissement Maillard)	2014	Larva	_	Engorged tick, collected on passerine	0
Amblyomma longirostre	_	Ixodidae	Macouria (Lotissement Maillard)	2014	Larva	_	Engorged tick, collected on passerine	0
Amblyomma longirostre	_	Ixodidae	Montsinéry (Chemin de Risquetout)	2014	Nymph	_	Engorged tick, collected on passerine	0
Amblyomma longirostre	_	Ixodidae	Montsinéry (Chemin de Risquetout)	2014	Larva	_	Engorged tick, collected on passerine	0
Amblyomma longirostre	_	Ixodidae	Matoury (Mont Paramana)	2014	Larva	_	Engorged tick, collected on passerine	0
Amblyomma longirostre	_	Ixodidae	Matoury (Mont Paramana)	2014	Larva	_	Engorged tick, collected on passerine	0
Amblyomma longirostre	_	Ixodidae	Matoury (Mont Paramana)	2014	Larva	_	Engorged tick, collected on passerine	0
Amblyomma longirostre	_	Ixodidae	Matoury (Mont Paramana)	2014	Larva	_	Engorged tick, collected on passerine	0
Amblyomma longirostre	_	Ixodidae	Matoury (Mont Paramana)	2014	Larva	_	Engorged tick, collected on passerine	0
Amblyomma longirostre	_	Ixodidae	Matoury (Mont Paramana)	2014	Nymph	_	Engorged tick, collected on passerine	0
Amblyomma longirostre	_	Ixodidae	Matoury (Mont Paramana)	2014	Larva	_	Engorged tick, collected on passerine	0
Amblyomma longirostre	_	Ixodidae	Matoury (Mont Paramana)	2014	Larva	_	Engorged tick, collected on passerine	0
Amblyomma longirostre	_	Ixodidae	Matoury (Mont Paramana)	2014	Larva	_	Engorged tick, collected on passerine	0
Amblyomma longirostre	_	Ixodidae	Matoury (Mont Paramana)	2014	Larva	_	Engorged tick, collected on passerine	0
Amblyomma longirostre	_	Ixodidae	Matoury (Mont Paramana)	2014	Larva	_	Engorged tick, collected on passerine	0
Amblyomma longirostre	_	Ixodidae	Matoury (Mont Paramana)	2014	Larva	_	Engorged tick, collected on passerine	0
Amblyomma longirostre	_	Ixodidae	Matoury (Mont Paramana)	2014	Larva	_	Engorged tick, collected on passerine	0
Amblyomma longirostre	_	Ixodidae	Matoury (Mont Paramana)	2014	Larva	_	Engorged tick, collected on passerine	0
Amblyomma longirostre	_	Ixodidae	Rémire-Montjoly (Vidal)	2013	Larva	_	Engorged tick, collected on passerine	0
Amblyomma longirostre	_	Ixodidae	Rémire-Montjoly (Vidal)	2013	Nymph	_	Engorged tick, collected on passerine	0
Amblyomma longirostre	_	Ixodidae	Rémire-Montjoly (Vidal)	2013	Larva	_	Engorged tick, collected on passerine	0
Amblyomma longirostre	_	Ixodidae	Rémire-Montjoly (Vidal)	2013	Larva	_	Engorged tick, collected on passerine	0
Amblyomma longirostre	_	Ixodidae	Rémire-Montjoly (Vidal)	2013	Larva	_	Engorged tick, collected on passerine	0
Amblyomma longirostre	_	Ixodidae	Rémire-Montjoly (Vidal)	2013	Larva	_	Engorged tick, collected on passerine	0
Amblyomma longirostre	_	Ixodidae	Rémire-Montjoly (Vidal)	2013	Larva	_	Engorged tick, collected on passerine	0
Amblyomma longirostre	_	Ixodidae	Rémire-Montjoly (Vidal)	2013	Larva	_	Engorged tick, collected on passerine	0
Amblyomma longirostre	_	Ixodidae	Rémire-Montjoly (Vidal)	2013	Larva	_	Engorged tick, collected on passerine	0
Amblyomma longirostre	_	Ixodidae	Rémire-Montjoly (Vidal)	2013	Larva	_	Engorged tick, collected on passerine	0
Amblyomma longirostre	_	Ixodidae	Rémire-Montjoly (Vidal)	2013	Larva	_	Engorged tick, collected on passerine	0
Amblyomma longirostre	_	Ixodidae	Rémire-Montjoly (Vidal)	2013	Larva	_	Engorged tick, collected on passerine	0
Amblyomma longirostre	_	Ixodidae	Rémire-Montjoly (Vidal)	2013	Larva	_	Engorged tick, collected on passerine	0
Amblyomma longirostre	_	Ixodidae	Rémire-Montjoly (Vidal)	2013	Larva	_	Engorged tick, collected on passerine	0
Amblyomma longirostre	_	Ixodidae	Rémire-Montjoly (Vidal)	2013	Larva	_	Engorged tick, collected on passerine	0
Amblyomma longirostre	_	Ixodidae	Rémire-Montjoly (Vidal)	2013	Larva	_	Engorged tick, collected on passerine	0
Amblyomma longirostre	_	Ixodidae	Rémire-Montjoly (Vidal)	2013	Larva	_	Engorged tick, collected on passerine	0
Amblyomma longirostre	_	Ixodidae	Saint-Georges-de-l'Oyapock (Ile Galion)	2018	Nymph	_	Engorged tick, collected on passerine	0
Amblyomma naponense	_	Ixodidae	Montagne de Kaw	2016	Nymph	_	Questing tick, collected on vegetation	0
Amblyomma naponense	_	Ixodidae	Montagne de Kaw	2016	Nymph	_	Questing tick, collected on vegetation	0
Amblyomma naponense	_	Ixodidae	Montagne de Kaw	2016	Nymph	_	Questing tick, collected on vegetation	0
Amblyomma naponense	_	Ixodidae	St-Georges de l'Oyapock (Crique Gabaret)	2016	Adult	Male	Questing tick, collected on vegetation	0
Amblyomma naponense	_	Ixodidae	Mana (Trinité)	2017	Adult	Female	Questing tick, collected on human	0
Amblyomma oblongoguttatum	_	Ixodidae	Kourou (CSG)	2016	Adult	Female	Questing tick, collected on human	0
Amblyomma oblongoguttatum	_	Ixodidae	Kourou (CSG)	2016	Adult	Female	Questing tick, collected on human	0
Amblyomma oblongoguttatum	_	Ixodidae	Kourou (CSG)	2016	Adult	Female	Questing tick, collected on human	0
Amblyomma oblongoguttatum	_	Ixodidae	Kourou (CSG)	2016	Adult	Female	Questing tick, collected on human	0
Amblyomma oblongoguttatum	_	Ixodidae	Kourou (CSG)	2016	Adult	Female	Questing tick, collected on human	0
Amblyomma oblongoguttatum	_	Ixodidae	Kourou (CSG)	2016	Adult	Female	Questing tick, collected on human	0
Amblyomma oblongoguttatum	_	Ixodidae	Kourou (CSG)	2016	Adult	Female	Questing tick, collected on human	0
Amblyomma oblongoguttatum	_	Ixodidae	Kourou (CSG)	2016	Adult	Female	Questing tick, collected on human	0
Amblyomma oblongoguttatum	_	Ixodidae	Kourou (CSG)	2016	Adult	Female	Questing tick, collected on human	0
Amblyomma oblongoguttatum	_	Ixodidae	Kourou (CSG)	2016	Adult	Female	Questing tick, collected on human	0
Amblyomma oblongoguttatum	_	Ixodidae	Kourou (CSG)	2016	Adult	Female	Questing tick, collected on human	1
Amblyomma oblongoguttatum	_	Ixodidae	Kourou (CSG)	2016	Adult	Female	Questing tick, collected on human	0
Amblyomma oblongoguttatum	_	Ixodidae	Kourou (CSG)	2016	Adult	Female	Questing tick, collected on human	0
Amblyomma oblongoguttatum	_	Ixodidae	Kourou (CSG)	2016	Adult	Female	Questing tick, collected on human	0
Amblyomma oblongoguttatum	_	Ixodidae	Kourou (CSG)	2016	Adult	Female	Questing tick, collected on human	0
Amblyomma oblongoguttatum	_	Ixodidae	Kourou (CSG)	2016	Adult	Female	Questing tick, collected on human	0
Amblyomma oblongoguttatum	_	Ixodidae	Kourou (CSG)	2016	Adult	Female	Questing tick, collected on human	0
Amblyomma oblongoguttatum	_	Ixodidae	Kourou (CSG)	2016	Adult	Female	Questing tick, collected on human	0
Amblyomma oblongoguttatum	_	Ixodidae	Kourou (CSG)	2016	Adult	Female	Questing tick, collected on human	0
Amblyomma oblongoguttatum	_	Ixodidae	Kourou (CSG)	2016	Adult	Female	Questing tick, collected on human	1
Amblyomma oblongoguttatum	_	Ixodidae	Kourou (CSG)	2016	Adult	Female	Questing tick, collected on human	0
Amblyomma oblongoguttatum	_	Ixodidae	Kourou (CSG)	2016	Adult	Female	Questing tick, collected on human	0
Amblyomma oblongoguttatum	_	Ixodidae	Kourou (CSG)	2016	Adult	Female	Questing tick, collected on human	0
Amblyomma oblongoguttatum	_	Ixodidae	Kourou (CSG)	2016	Adult	Female	Questing tick, collected on human	0
Amblyomma oblongoguttatum	_	Ixodidae	Kourou (CSG)	2016	Adult	Female	Questing tick, collected on human	0
Amblyomma oblongoguttatum	_	Ixodidae	Kourou (CSG)	2016	Adult	Female	Questing tick, collected on human	0
Amblyomma oblongoguttatum	_	Ixodidae	Kourou (CSG)	2016	Adult	Female	Questing tick, collected on human	0
Amblyomma oblongoguttatum	_	Ixodidae	Kourou (CSG)	2016	Adult	Female	Questing tick, collected on human	0
Amblyomma oblongoguttatum	_	Ixodidae	Kourou (CSG)	2016	Adult	Female	Questing tick, collected on human	0
Amblyomma oblongoguttatum	_	Ixodidae	Kourou (CSG)	2016	Adult	Female	Questing tick, collected on human	0
Amblyomma oblongoguttatum	_	Ixodidae	Kourou (CSG)	2016	Adult	Male	Questing tick, collected on human	0
Amblyomma oblongoguttatum	_	Ixodidae	Kourou (CSG)	2016	Adult	Male	Questing tick, collected on human	0
Amblyomma oblongoguttatum	_	Ixodidae	Kourou (CSG)	2016	Adult	Male	Questing tick, collected on human	0
Amblyomma oblongoguttatum	_	Ixodidae	Kourou (CSG)	2016	Adult	Male	Questing tick, collected on human	0
Amblyomma oblongoguttatum	_	Ixodidae	Kourou (CSG)	2016	Adult	Male	Questing tick, collected on human	0
Amblyomma oblongoguttatum	_	Ixodidae	Kourou (CSG)	2016	Adult	Male	Questing tick, collected on human	0
Amblyomma oblongoguttatum	_	Ixodidae	Kourou (CSG)	2016	Adult	Male	Questing tick, collected on human	0
Amblyomma oblongoguttatum	_	Ixodidae	Kourou (CSG)	2016	Adult	Male	Questing tick, collected on human	0
Amblyomma oblongoguttatum	_	Ixodidae	Kourou (CSG)	2016	Adult	Male	Questing tick, collected on human	0
Amblyomma oblongoguttatum	_	Ixodidae	Kourou (CSG)	2016	Adult	Male	Questing tick, collected on human	0
Amblyomma oblongoguttatum	_	Ixodidae	Kourou (CSG)	2016	Adult	Male	Questing tick, collected on human	0
Amblyomma oblongoguttatum	_	Ixodidae	Kourou (CSG)	2016	Adult	Male	Questing tick, collected on human	0
Amblyomma oblongoguttatum	_	Ixodidae	Kourou (CSG)	2016	Adult	Male	Questing tick, collected on human	0
Amblyomma oblongoguttatum	_	Ixodidae	Kourou (CSG)	2016	Adult	Male	Questing tick, collected on human	0
Amblyomma oblongoguttatum	_	Ixodidae	Kourou (CSG)	2016	Adult	Male	Questing tick, collected on human	0
Amblyomma oblongoguttatum	_	Ixodidae	Kourou (CSG)	2016	Adult	Male	Questing tick, collected on human	0
Amblyomma oblongoguttatum	_	Ixodidae	Kourou (CSG)	2016	Adult	Male	Questing tick, collected on human	0
Amblyomma oblongoguttatum	_	Ixodidae	Kourou (CSG)	2016	Adult	Male	Questing tick, collected on human	0
Amblyomma oblongoguttatum	_	Ixodidae	Kourou (CSG)	2016	Adult	Male	Questing tick, collected on human	0
Amblyomma oblongoguttatum	_	Ixodidae	Kourou (CSG)	2016	Adult	Male	Questing tick, collected on human	0
Amblyomma oblongoguttatum	_	Ixodidae	Kourou (CSG)	2016	Adult	Male	Questing tick, collected on human	0
Amblyomma oblongoguttatum	_	Ixodidae	Kourou (CSG)	2016	Adult	Male	Questing tick, collected on human	0
Amblyomma oblongoguttatum	_	Ixodidae	Kourou (CSG)	2016	Adult	Male	Questing tick, collected on human	0
Amblyomma oblongoguttatum	_	Ixodidae	Kourou (CSG)	2016	Adult	Male	Questing tick, collected on human	0
Amblyomma oblongoguttatum	_	Ixodidae	Kourou (CSG)	2016	Adult	Male	Questing tick, collected on human	0
Amblyomma oblongoguttatum	_	Ixodidae	Kourou (CSG)	2016	Adult	Male	Questing tick, collected on human	0
Amblyomma oblongoguttatum	_	Ixodidae	Kourou (CSG)	2016	Adult	Male	Questing tick, collected on human	0
Amblyomma oblongoguttatum	_	Ixodidae	Kourou (CSG)	2016	Adult	Male	Questing tick, collected on human	0
Amblyomma oblongoguttatum	_	Ixodidae	Kourou (CSG)	2016	Adult	Male	Questing tick, collected on human	0

Amblyomma oblongoguttatum	_	Ixodidae	Kourou (CSG)	2016	Adult	Male	Questing tick, collected on human	0
Amblyomma oblongoguttatum	_	Ixodidae	Kourou (CSG)	2016	Adult	Male	Questing tick, collected on human	0
Amblyomma oblongoguttatum	_	Ixodidae	Kourou (CSG)	2016	Adult	Male	Questing tick, collected on human	0
Amblyomma oblongoguttatum	_	Ixodidae	Kourou (CSG)	2016	Adult	Male	Questing tick, collected on human	0
Amblyomma oblongoguttatum	_	Ixodidae	Kourou (CSG)	2016	Adult	Male	Questing tick, collected on human	0
Amblyomma oblongoguttatum	_	Ixodidae	Kourou (CSG)	2016	Adult	Male	Questing tick, collected on human	0
Amblyomma oblongoguttatum	_	Ixodidae	Kourou (CSG)	2016	Adult	Male	Questing tick, collected on human	0
Amblyomma pacae	_	Ixodidae	St-Georges de l'Oyapock (Crique Gabaret)	2016	Larva	_	Questing tick, collected on vegetation	0
Amblyomma pacae	_	Ixodidae	St-Georges de l'Oyapock (Crique Gabaret)	2016	Larva	_	Questing tick, collected on vegetation	0
Amblyomma pacae	_	Ixodidae	St-Georges de l'Oyapock (Crique Gabaret)	2016	Larva	_	Questing tick, collected on vegetation	0
Amblyomma pacae	_	Ixodidae	St-Georges de l'Oyapock (Crique Gabaret)	2016	Larva	_	Questing tick, collected on vegetation	0
Amblyomma pacae	_	Ixodidae	St-Georges de l'Oyapock (Crique Gabaret)	2016	Larva	_	Questing tick, collected on vegetation	0
Amblyomma pacae	_	Ixodidae	St-Georges de l'Oyapock (Crique Gabaret)	2016	Larva	_	Questing tick, collected on vegetation	0
Amblyomma romitii	_	Ixodidae	Macouria (Tonate)	2017	Adult	Female	Engorged tick, collected on capybara	1
Amblyomma romitii	_	Ixodidae	Macouria (Tonate)	2017	Adult	Female	Engorged tick, collected on capybara	0
Amblyomma rotundatum	_	Ixodidae	Kourou (Montagne des Singes)	2016	Larva	_	Questing tick, collected on vegetation	0
Amblyomma rotundatum	_	Ixodidae	Kourou (Montagne des Singes)	2016	Larva	_	Questing tick, collected on vegetation	0
Amblyomma rotundatum	_	Ixodidae	Kourou (Montagne des Singes)	2016	Larva	_	Questing tick, collected on vegetation	0
Amblyomma rotundatum	_	Ixodidae	Kourou (Montagne des Singes)	2016	Larva	_	Questing tick, collected on vegetation	0
Amblyomma rotundatum	_	Ixodidae	Kourou (Montagne des Singes)	2016	Larva	_	Questing tick, collected on vegetation	0
Amblyomma rotundatum	_	Ixodidae	Iracoubo (Counamie)	2017	Adult	Female	Engorged tick, collected on tortoise	0
Amblyomma scalpturatum	_	Ixodidae	Kourou (Montagne des Singes)	2016	Adult	Female	Questing tick, collected on vegetation	0
Amblyomma scalpturatum	_	Ixodidae	Régina (Savane-Roche Virginie)	2016	Adult	Female	Questing tick, collected on vegetation	0
Amblyomma scalpturatum	_	Ixodidae	Kourou (CSG)	2016	Nymph	_	Questing tick, collected on human	0
Amblyomma scalpturatum	_	Ixodidae	Kourou (CSG)	2016	Nymph	_	Questing tick, collected on human	0
Amblyomma scalpturatum	_	Ixodidae	Kourou (CSG)	2016	Nymph	_	Questing tick, collected on human	0
Amblyomma scalpturatum	_	Ixodidae	Petit-Saut (Montagne Plomb)	2016	Adult	Male	Questing tick, collected on human	0
Amblyomma scalpturatum	_	Ixodidae	Nouragues	2017	Larva	_	Questing tick, collected on human	0
Amblyomma scalpturatum	_	Ixodidae	Nouragues	2017	Larva	_	Questing tick, collected on human	0
Amblyomma scalpturatum	_	Ixodidae	Nouragues	2017	Larva	_	Questing tick, collected on human	0
Amblyomma varium	Sloth's giant tick	Ixodidae	Matoury (Larivot)	2016	Adult	Male	Engorged tick, collected on sloth	0
Amblyomma varium	Sloth's giant tick	Ixodidae	Matoury (Larivot)	2016	Adult	Male	Engorged tick, collected on sloth	0
Amblyomma varium	Sloth's giant tick	Ixodidae	Rémire-Montjoly (Vidal)	2016	Adult	Female	Engorged tick, collected on sloth	0
Amblyomma varium	Sloth's giant tick	Ixodidae	Matoury (Piste de la Mirande)	2017	Larva	_	Questing tick, collected on vegetation	0
Amblyomma varium	Sloth's giant tick	Ixodidae	Matoury (Piste de la Mirande)	2017	Larva	_	Questing tick, collected on vegetation	0
Amblyomma varium	Sloth's giant tick	Ixodidae	Matoury (Piste de la Mirande)	2017	Larva	_	Questing tick, collected on vegetation	0
Amblyomma varium	Sloth's giant tick	Ixodidae	Rémire-Montjloy (Sentier Loyola)	2017	Nymph	_	Questing tick, collected on vegetation	0
Rhipicephalus microplus	Asian blue tick/Tropical cattle tick	Ixodidae	Macouria (Matiti)	2016	Adult	Male	Engorged tick, collected on cattle	0
Rhipicephalus microplus	Asian blue tick/Tropical cattle tick	Ixodidae	Macouria (Matiti)	2016	Adult	Male	Engorged tick, collected on cattle	0
Rhipicephalus microplus	Asian blue tick/Tropical cattle tick	Ixodidae	Macouria (Matiti)	2016	Adult	Male	Engorged tick, collected on cattle	0
Rhipicephalus microplus	Asian blue tick/Tropical cattle tick	Ixodidae	Macouria (Matiti)	2016	Adult	Male	Engorged tick, collected on cattle	0
Rhipicephalus microplus	Asian blue tick/Tropical cattle tick	Ixodidae	Macouria (Matiti)	2016	Adult	Male	Engorged tick, collected on cattle	0
Rhipicephalus microplus	Asian blue tick/Tropical cattle tick	Ixodidae	Macouria (Matiti)	2016	Adult	Male	Engorged tick, collected on cattle	0
Rhipicephalus microplus	Asian blue tick/Tropical cattle tick	Ixodidae	Macouria (Matiti)	2016	Larva	_	Questing tick, collected on vegetation	0
Rhipicephalus microplus	Asian blue tick/Tropical cattle tick	Ixodidae	Macouria (Matiti)	2016	Larva	_	Questing tick, collected on vegetation	0
Rhipicephalus microplus	Asian blue tick/Tropical cattle tick	Ixodidae	Macouria (Matiti)	2016	Larva	_	Questing tick, collected on vegetation	0
Rhipicephalus microplus	Asian blue tick/Tropical cattle tick	Ixodidae	Macouria (Matiti)	2016	Larva	_	Questing tick, collected on vegetation	0
Rhipicephalus sanguineus	Brown dog tick	Ixodidae	Kourou (Rue Maurice Ravel)	2016	Adult	Female	Engorged tick, collected on domestic dog	0
Rhipicephalus sanguineus	Brown dog tick	Ixodidae	Kourou (Rue Maurice Ravel)	2016	Adult	Female	Engorged tick, collected on domestic dog	0
Rhipicephalus sanguineus	Brown dog tick	Ixodidae	Kourou (Rue Maurice Ravel)	2016	Adult	Female	Engorged tick, collected on domestic dog	0
Rhipicephalus sanguineus	Brown dog tick	Ixodidae	Rémire-Montjoly (Montravel)	2016	Adult	Male	Questing tick, collected on domestic dog	0
Rhipicephalus sanguineus	Brown dog tick	Ixodidae	Rémire-Montjoly (Montravel)	2016	Adult	Male	Questing tick, collected on domestic dog	0
Rhipicephalus sanguineus	Brown dog tick	Ixodidae	Rémire-Montjoly (Montravel)	2016	Adult	Male	Questing tick, collected on domestic dog	1
Dermacentor nitens	Tropical horse tick	Ixodidae	Macouria (Ranch Le Papayer)	2016	Adult	Female	Engorged tick, collected on domestic horse	0
Dermacentor nitens	Tropical horse tick	Ixodidae	Macouria (Ranch Le Papayer)	2016	Adult	Female	Engorged tick, collected on domestic horse	0
Dermacentor nitens	Tropical horse tick	Ixodidae	Macouria (Ranch Le Papayer)	2016	Adult	Male	Engorged tick, collected on domestic horse	0
Dermacentor nitens	Tropical horse tick	Ixodidae	Macouria (Ranch Le Papayer)	2016	Adult	Male	Engorged tick, collected on domestic horse	0
Dermacentor nitens	Tropical horse tick	Ixodidae	Macouria (Ranch Le Papayer)	2016	Nymph	_	Engorged tick, collected on domestic horse	0
Dermacentor nitens	Tropical horse tick	Ixodidae	Macouria (Ranch Le Papayer)	2016	Nymph	_	Engorged tick, collected on domestic horse	0
Dermacentor nitens	Tropical horse tick	Ixodidae	Macouria (Ranch Le Papayer)	2016	Adult	Female	Engorged tick, collected on domestic horse	0
Dermacentor nitens	Tropical horse tick	Ixodidae	Macouria (Ranch Le Papayer)	2016	Adult	Female	Engorged tick, collected on domestic horse	0
Dermacentor nitens	Tropical horse tick	Ixodidae	Macouria (Ranch Le Papayer)	2016	Adult	Female	Engorged tick, collected on domestic horse	0
Dermacentor nitens	Tropical horse tick	Ixodidae	Macouria (Ranch Le Papayer)	2016	Adult	Female	Engorged tick, collected on domestic horse	0
Dermacentor nitens	Tropical horse tick	Ixodidae	Macouria (Ranch Le Papayer)	2016	Adult	Female	Engorged tick, collected on domestic horse	0
Dermacentor nitens	Tropical horse tick	Ixodidae	Macouria (Ranch Le Papayer)	2016	Adult	Female	Engorged tick, collected on domestic horse	0
Dermacentor nitens	Tropical horse tick	Ixodidae	Macouria (Ranch Le Papayer)	2016	Adult	Male	Engorged tick, collected on domestic horse	0
Dermacentor nitens	Tropical horse tick	Ixodidae	Macouria (Ranch Le Papayer)	2016	Adult	Male	Engorged tick, collected on domestic horse	0
Dermacentor nitens	Tropical horse tick	Ixodidae	Macouria (Ranch Le Papayer)	2016	Adult	Male	Engorged tick, collected on domestic horse	0
Dermacentor nitens	Tropical horse tick	Ixodidae	Macouria (Ranch Le Papayer)	2016	Adult	Male	Engorged tick, collected on domestic horse	0
Dermacentor nitens	Tropical horse tick	Ixodidae	Macouria (Ranch Le Papayer)	2016	Adult	Male	Engorged tick, collected on domestic horse	0
Dermacentor nitens	Tropical horse tick	Ixodidae	Macouria (Ranch Le Papayer)	2016	Nymph	_	Engorged tick, collected on domestic horse	0
Dermacentor nitens	Tropical horse tick	Ixodidae	Macouria (Ranch Le Papayer)	2016	Nymph	_	Engorged tick, collected on domestic horse	0
Dermacentor nitens	Tropical horse tick	Ixodidae	Macouria (Ranch Le Papayer)	2016	Nymph	_	Engorged tick, collected on domestic horse	0
Dermacentor nitens	Tropical horse tick	Ixodidae	Macouria (Ranch Le Papayer)	2016	Nymph	_	Engorged tick, collected on domestic horse	0
Dermacentor nitens	Tropical horse tick	Ixodidae	Macouria (Ranch Le Papayer)	2016	Nymph	_	Engorged tick, collected on domestic horse	0
Dermacentor nitens	Tropical horse tick	Ixodidae	Macouria (Ranch Le Papayer)	2016	Nymph	_	Engorged tick, collected on domestic horse	0
Dermacentor nitens	Tropical horse tick	Ixodidae	Macouria (Ranch Le Papayer)	2016	Nymph	_	Engorged tick, collected on domestic horse	0
Dermacentor nitens	Tropical horse tick	Ixodidae	Macouria (Ranch Le Papayer)	2016	Nymph	_	Engorged tick, collected on domestic horse	0
Dermacentor nitens	Tropical horse tick	Ixodidae	Macouria (Ranch Le Papayer)	2016	Nymph	_	Engorged tick, collected on domestic horse	0
Dermacentor nitens	Tropical horse tick	Ixodidae	Macouria (Ranch Le Papayer)	2016	Nymph	_	Engorged tick, collected on domestic horse	0
Dermacentor nitens	Tropical horse tick	Ixodidae	Macouria (Ranch Le Papayer)	2016	Nymph	_	Engorged tick, collected on domestic horse	0
Dermacentor nitens	Tropical horse tick	Ixodidae	Macouria (Ranch Le Papayer)	2016	Nymph	_	Engorged tick, collected on domestic horse	0
Dermacentor nitens	Tropical horse tick	Ixodidae	Macouria (Ranch Le Papayer)	2016	Nymph	_	Engorged tick, collected on domestic horse	0
Dermacentor nitens	Tropical horse tick	Ixodidae	Macouria (Ranch Le Papayer)	2016	Nymph	_	Engorged tick, collected on domestic horse	0
Dermacentor nitens	Tropical horse tick	Ixodidae	Macouria (Ranch Le Papayer)	2016	Nymph	_	Engorged tick, collected on domestic horse	0
Dermacentor nitens	Tropical horse tick	Ixodidae	Macouria (Ranch Le Papayer)	2016	Larva	_	Engorged tick, collected on domestic horse	0
Dermacentor nitens	Tropical horse tick	Ixodidae	Macouria (Ranch Le Papayer)	2016	Larva	_	Engorged tick, collected on domestic horse	0
Dermacentor nitens	Tropical horse tick	Ixodidae	Macouria (Ranch Le Papayer)	2016	Larva	_	Engorged tick, collected on domestic horse	0
Dermacentor nitens	Tropical horse tick	Ixodidae	Macouria (Ranch Le Papayer)	2016	Larva	_	Engorged tick, collected on domestic horse	0
Dermacentor nitens	Tropical horse tick	Ixodidae	Macouria (Ranch Le Papayer)	2016	Larva	_	Engorged tick, collected on domestic horse	0
Dermacentor nitens	Tropical horse tick	Ixodidae	Macouria (Ranch Le Papayer)	2016	Larva	_	Engorged tick, collected on domestic horse	0
Dermacentor nitens	Tropical horse tick	Ixodidae	Macouria (Ranch Le Papayer)	2016	Larva	_	Engorged tick, collected on domestic horse	0
Dermacentor nitens	Tropical horse tick	Ixodidae	Macouria (Ranch Le Papayer)	2016	Larva	_	Engorged tick, collected on domestic horse	0
Dermacentor nitens	Tropical horse tick	Ixodidae	Macouria (Ranch Le Papayer)	2016	Larva	_	Engorged tick, collected on domestic horse	0
Dermacentor nitens	Tropical horse tick	Ixodidae	Macouria (Ranch Le Papayer)	2016	Larva	_	Engorged tick, collected on domestic horse	0
Dermacentor nitens	Tropical horse tick	Ixodidae	Macouria (Ranch Le Papayer)	2016	Larva	_	Engorged tick, collected on domestic horse	0
Dermacentor nitens	Tropical horse tick	Ixodidae	Macouria (Ranch Le Papayer)	2016	Larva	_	Engorged tick, collected on domestic horse	0
Dermacentor nitens	Tropical horse tick	Ixodidae	Macouria (Ranch Le Papayer)	2016	Larva	_	Engorged tick, collected on domestic horse	0
Dermacentor nitens	Tropical horse tick	Ixodidae	Macouria (Ranch Le Papayer)	2016	Larva	_	Engorged tick, collected on domestic horse	0
Dermacentor nitens	Tropical horse tick	Ixodidae	Macouria (Ranch Le Papayer)	2016	Adult	Female	Engorged tick, collected on domestic horse	0
Dermacentor nitens	Tropical horse tick	Ixodidae	Macouria (Ranch Le Papayer)	2016	Adult	Female	Engorged tick, collected on domestic horse	0
Dermacentor nitens	Tropical horse tick	Ixodidae	Macouria (Ranch Le Papayer)	2016	Adult	Female	Engorged tick, collected on domestic horse	0
Dermacentor nitens	Tropical horse tick	Ixodidae	Macouria (Ranch Le Papayer)	2016	Adult	Female	Engorged tick, collected on domestic horse	0
Dermacentor nitens	Tropical horse tick	Ixodidae	Macouria (Ranch Le Papayer)	2016	Adult	Female	Engorged tick, collected on domestic horse	0
Dermacentor nitens	Tropical horse tick	Ixodidae	Macouria (Ranch Le Papayer)	2016	Adult	Male	Engorged tick, collected on domestic horse	0
Dermacentor nitens	Tropical horse tick	Ixodidae	Macouria (Ranch Le Papayer)	2016	Adult	Male	Engorged tick, collected on domestic horse	0
Dermacentor nitens	Tropical horse tick	Ixodidae	Macouria (Ranch Le Papayer)	2016	Adult	Male	Engorged tick, collected on domestic horse	0
Dermacentor nitens	Tropical horse tick	Ixodidae	Macouria (Ranch Le Papayer)	2016	Adult	Male	Engorged tick, collected on domestic horse	0
Dermacentor nitens	Tropical horse tick	Ixodidae	Macouria (Ranch Le Papayer)	2016	Adult	Male	Engorged tick, collected on domestic horse	0
Dermacentor nitens	Tropical horse tick	Ixodidae	Macouria (Ranch Le Papayer)	2016	Nymph	_	Engorged tick, collected on domestic horse	0
Dermacentor nitens	Tropical horse tick	Ixodidae	Macouria (Ranch Le Papayer)	2016	Nymph	_	Engorged tick, collected on domestic horse	0
Dermacentor nitens	Tropical horse tick	Ixodidae	Macouria (Ranch Le Papayer)	2016	Nymph	_	Engorged tick, collected on domestic horse	0
Dermacentor nitens	Tropical horse tick	Ixodidae	Macouria (Ranch Le Papayer)	2016	Nymph	_	Engorged tick, collected on domestic horse	0
Dermacentor nitens	Tropical horse tick	Ixodidae	Macouria (Ranch Le Papayer)	2016	Adult	Male	Engorged tick, collected on domestic horse	0
Dermacentor nitens	Tropical horse tick	Ixodidae	Macouria (Ranch Le Papayer)	2016	Adult	Male	Engorged tick, collected on domestic horse	0
Dermacentor nitens	Tropical horse tick	Ixodidae	Macouria (Ranch Le Papayer)	2016	Adult	Male	Engorged tick, collected on domestic horse	0
Dermacentor nitens	Tropical horse tick	Ixodidae	Macouria (Ranch Le Papayer)	2016	Nymph	_	Engorged tick, collected on domestic horse	0
Dermacentor nitens	Tropical horse tick	Ixodidae	Macouria (Ranch Le Papayer)	2016	Nymph	_	Engorged tick, collected on domestic horse	0
Dermacentor nitens	Tropical horse tick	Ixodidae	Macouria (Ranch Le Papayer)	2016	Nymph	_	Engorged tick, collected on domestic horse	0
Dermacentor nitens	Tropical horse tick	Ixodidae	Macouria (Ranch Le Papayer)	2016	Nymph	_	Engorged tick, collected on domestic horse	0
Dermacentor nitens	Tropical horse tick	Ixodidae	Macouria (Ranch Le Papayer)	2016	Nymph	_	Engorged tick, collected on domestic horse	0
Dermacentor nitens	Tropical horse tick	Ixodidae	Macouria (Ranch Le Papayer)	2016	Nymph	_	Engorged tick, collected on domestic horse	0
Dermacentor nitens	Tropical horse tick	Ixodidae	Macouria (Ranch Le Papayer)	2016	Larva	_	Engorged tick, collected on domestic horse	0
Dermacentor nitens	Tropical horse tick	Ixodidae	Macouria (Ranch Le Papayer)	2016	Larva	_	Engorged tick, collected on domestic horse	0
Dermacentor nitens	Tropical horse tick	Ixodidae	Macouria (Ranch Le Papayer)	2016	Larva	_	Engorged tick, collected on domestic horse	0
Dermacentor nitens	Tropical horse tick	Ixodidae	Macouria (Ranch Le Papayer)	2016	Larva	_	Engorged tick, collected on domestic horse	0
Dermacentor nitens	Tropical horse tick	Ixodidae	Macouria (Ranch Le Papayer)	2016	Larva	_	Engorged tick, collected on domestic horse	0
Dermacentor nitens	Tropical horse tick	Ixodidae	Macouria (Ranch Le Papayer)	2016	Larva	_	Engorged tick, collected on domestic horse	0
Dermacentor nitens	Tropical horse tick	Ixodidae	Macouria (Ranch Le Papayer)	2016	Larva	_	Engorged tick, collected on domestic horse	0
Dermacentor nitens	Tropical horse tick	Ixodidae	Macouria (Ranch Le Papayer)	2016	Larva	_	Engorged tick, collected on domestic horse	0
Dermacentor nitens	Tropical horse tick	Ixodidae	Macouria (Ranch Le Papayer)	2016	Adult	Female	Engorged tick, collected on domestic horse	0
Dermacentor nitens	Tropical horse tick	Ixodidae	Macouria (Ranch Le Papayer)	2016	Adult	Female	Engorged tick, collected on domestic horse	0

Dermacentor nitens	Tropical horse tick	Ixodidae	Macouria (Ranch Le Papayer)	2016	Adult	Female	Engorged tick, collected on domestic horse	0
Dermacentor nitens	Tropical horse tick	Ixodidae	Macouria (Ranch Le Papayer)	2016	Adult	Female	Engorged tick, collected on domestic horse	0
Dermacentor nitens	Tropical horse tick	Ixodidae	Macouria (Ranch Le Papayer)	2016	Adult	Female	Engorged tick, collected on domestic horse	0
Dermacentor nitens	Tropical horse tick	Ixodidae	Macouria (Ranch Le Papayer)	2016	Adult	Male	Engorged tick, collected on domestic horse	0
Dermacentor nitens	Tropical horse tick	Ixodidae	Macouria (Ranch Le Papayer)	2016	Adult	Male	Engorged tick, collected on domestic horse	0
Dermacentor nitens	Tropical horse tick	Ixodidae	Macouria (Ranch Le Papayer)	2016	Adult	Male	Engorged tick, collected on domestic horse	0
Dermacentor nitens	Tropical horse tick	Ixodidae	Macouria (Ranch Le Papayer)	2016	Adult	Male	Engorged tick, collected on domestic horse	0
Dermacentor nitens	Tropical horse tick	Ixodidae	Macouria (Ranch Le Papayer)	2016	Adult	Male	Engorged tick, collected on domestic horse	0
Dermacentor nitens	Tropical horse tick	Ixodidae	Macouria (Ranch Le Papayer)	2016	Nymph	_	Engorged tick, collected on domestic horse	0
Dermacentor nitens	Tropical horse tick	Ixodidae	Macouria (Ranch Le Papayer)	2016	Nymph	_	Engorged tick, collected on domestic horse	0
Dermacentor nitens	Tropical horse tick	Ixodidae	Macouria (Ranch Le Papayer)	2016	Nymph	_	Engorged tick, collected on domestic horse	0
Dermacentor nitens	Tropical horse tick	Ixodidae	Macouria (Ranch Le Papayer)	2016	Nymph	_	Engorged tick, collected on domestic horse	0
Dermacentor nitens	Tropical horse tick	Ixodidae	Macouria (Ranch Le Papayer)	2016	Nymph	_	Engorged tick, collected on domestic horse	0
Dermacentor nitens	Tropical horse tick	Ixodidae	Macouria (Ranch Le Papayer)	2016	Larva	_	Engorged tick, collected on domestic horse	0
Dermacentor nitens	Tropical horse tick	Ixodidae	Macouria (Ranch Le Papayer)	2016	Larva	_	Engorged tick, collected on domestic horse	0
Dermacentor nitens	Tropical horse tick	Ixodidae	Macouria (Ranch Le Papayer)	2016	Larva	_	Engorged tick, collected on domestic horse	0
Dermacentor nitens	Tropical horse tick	Ixodidae	Macouria (Ranch Le Papayer)	2016	Larva	_	Engorged tick, collected on domestic horse	0
Dermacentor nitens	Tropical horse tick	Ixodidae	Macouria (Ranch Le Papayer)	2016	Larva	_	Engorged tick, collected on domestic horse	0
Haemaphysalis juxtakochi	_	Ixodidae	Montagne Favard	2016	Nymph	_	Questing tick, collected on vegetation	0
Haemaphysalis juxtakochi	_	Ixodidae	Kourou (Montagne des Singes)	2016	Nymph	_	Questing tick, collected on vegetation	0
Haemaphysalis juxtakochi	_	Ixodidae	Kourou (Montagne des Singes)	2016	Nymph	_	Questing tick, collected on vegetation	0
Haemaphysalis juxtakochi	_	Ixodidae	Petit-Saut (Montagne Plomb)	2016	Nymph	_	Questing tick, collected on vegetation	0
Haemaphysalis juxtakochi	_	Ixodidae	Petit-Saut (Montagne Plomb)	2016	Nymph	_	Questing tick, collected on vegetation	0
Haemaphysalis juxtakochi	_	Ixodidae	Petit-Saut (Montagne Plomb)	2016	Nymph	_	Questing tick, collected on vegetation	0
Haemaphysalis juxtakochi	_	Ixodidae	Petit-Saut (Montagne Plomb)	2016	Nymph	_	Questing tick, collected on vegetation	0
Haemaphysalis juxtakochi	_	Ixodidae	Petit-Saut (Montagne Plomb)	2016	Nymph	_	Questing tick, collected on vegetation	0
Ixodes luciae	_	Ixodidae	Rémire-Montjoly (Vidal)	2016	Nymph	_	Engorged tick, collected on opossum	0
Ixodes luciae	_	Ixodidae	Cayenne (Montagne du Tigre)	2017	Adult	Female	Engorged tick, collected on opossum	0
Ixodes luciae	_	Ixodidae	Cayenne (Montagne du Tigre)	2017	Adult	Female	Engorged tick, collected on opossum	1
Ixodes luciae	_	Ixodidae	Cayenne (Montagne du Tigre)	2017	Adult	Male	Engorged tick, collected on opossum	1
Ixodes luciae	_	Ixodidae	Cayenne (Montagne du Tigre)	2017	Adult	Male	Engorged tick, collected on opossum	0
Ixodes luciae	_	Ixodidae	Cayenne (Montagne du Tigre)	2017	Larva	_	Engorged tick, collected on opossum	0
